# Supplementary material for: A common mechanism for recruiting the Rrm3 and RTEL1 accessory helicases to the eukaryotic replisome
Source: EMBO J. 2024 Jul 22;43(18):3. doi: 10.1038/s44318-024-00168-4 (PMC11405395; doi:10.1038/s44318-024-00168-4)
Supplement: Supplementary file 1 — Appendix [file 44318_2024_168_MOESM1_ESM.pdf]

Appendix for

A common mechanism for recruiting the Rrm3 and RTEL1 accessory helicases to the eukaryotic replisome

Ottavia Olson[1], Simone Pelliciari[1,2], Emma D. Heron[1,2] & Tom D. Deegan[1,*]

1 MRC Human Genetics Unit, Institute of Genetics and Cancer, University of Edinburgh, Western General Hospital, Edinburgh, EH4 2XU, U.K.
2 These authors contributed equally to this work

*Correspondence: tdeegan@ed.ac.uk

Table of Contents

Appendix Table S1 page 2
Appendix Table S2 page 3
Appendix Figure S1 page 5
Appendix Figure S2 page 6

Partner (Rrm3)	avg_n_models	max_n_models	best_pdockq	best_plddt_avg	best_pae_avg
Cdc45	0	0	0	0	0
Ctf4	0	0	0	0	0
Dpb2	3	5	0.741	74.6	6.9
Dpb3-4	0	0	0	0	0
GINS	3.4	4	0.544	80.2	5.4
Mcm2	0	0	0	0	0
Mcm3	0	0	0	0	0
Mcm4	0	0	0	0	0
Mcm5	0	0	0	0	0
Mcm6	0	0	0	0	0
Mcm7	0	0	0	0	0
Mcm10	0	0	0	0	0
Mrc1	0	0	0	0	0
PCNA	1.9	2	0.741	73.7	5.4
Pol1	0	0	0	0	0
Pol2	0	0	0	0	0
Pol12	0	0	0	0	0
Pri1-Pri2	0	0	0	0	0
RPA	0	0	0	0	0
Tof1-Csm3	0	0	0	0	0

Appendix Table S1. AlphaFold Multimer screening results for Rrm3 replisome interactors in S. cerevisiae. The results of folding Rrm3 with 23 replisome components are tabulated with 5 confidence metrics (see Legend for descriptions of metrics).

Pair	avg_n_models	max_n_models	best_pdockq	best_plddt_avg	best_pae_avg
DDX3_GINS	0	0	0	0	0
DDX11_GINS	0	0	0	0	0
FANCJ_GINS	0	0	0	0	0
HELB_GINS	1	1	0.018	58.9	12.7
PIF1_GINS	0	0	0	0	0
RTEL1_GINS	2.5	5	0.742	80.2	5.2
XPD_GINS	1.8	2	0.296	82	10.2
DDX3_POLE2	0	0	0	0	0
DDX11_POLE2	0	0	0	0	0
FANCJ_POLE2	0	0	0	0	0
HELB_POLE2	0	0	0	0	0
PIF1_POLE2	1	1	0.424	71	11.3
RTEL1_POLE2	4.4	5	0.738	80.9	4.9
XPD_POLE2	0	0	0	0	0

Appendix Table S2. AlphaFold Multimer screening results for Homo sapiens 5'-3' helicases with POLE2 and GINS. The results of folding seven human 5'-3' helicases with POLE2 or GINS are tabulated with 5 confidence metrics (see Legend for descriptions of metrics). The HELB-GINS interaction predicted in a single model was not followed up due to the very low pDockQ score. Inspection of the PIF1-POLE2 interaction predicted in a single model revealed extensive steric clashes at the PIF1-POLE2 interface and this was therefore not investigated further. An interface between XPD and GINS was predicted in two out of five models, although the pDockQ and best_pae_avg values are close to the threshold values (pDockQ > 0.25, best_pae_avg < 15) used to distinguish high confidence predictions.

Metric	Definition
avg_n_models	The average number of models that predict the same interface for a given protein pair.
max_n_models	The maximum number of models that satisfy at least some of the contacts in a predicted protein pair. The maximum possible value is equal to the total number of models generated by AlphaFold Multimer (five in this study).
best_model_avg_plddt	The predicted local distance difference test (pLDDT) values range from 0 (worst) to 100 (best), and describe the confidence that a specific amino acid is positioned correctly relative to neighbouring amino acids. Best_model_avg_plddt describes the average pLDDT of all the residues at the protein-protein interaction interface in the best model. High confidence predictions generally have values >70.
best_model_pdockq	Best_model_pdockq ranges from 0 (worst) to 1 (best) and takes into account the number of interacting residues and the average pLDDT of all those residues, in the highest confidence model. High confidence predictions generally have a value > 0.25.
best_model_avg_pae	The predicted alignment error (PAE, measured in angstroms) ranges from 0 (best) to 30 (worst) and is a measure of the predicted error in the relative positioning of two residues in a given structure. Best_model_avg_pae gives the average value for all interacting residue pairs for the highest confidence model.

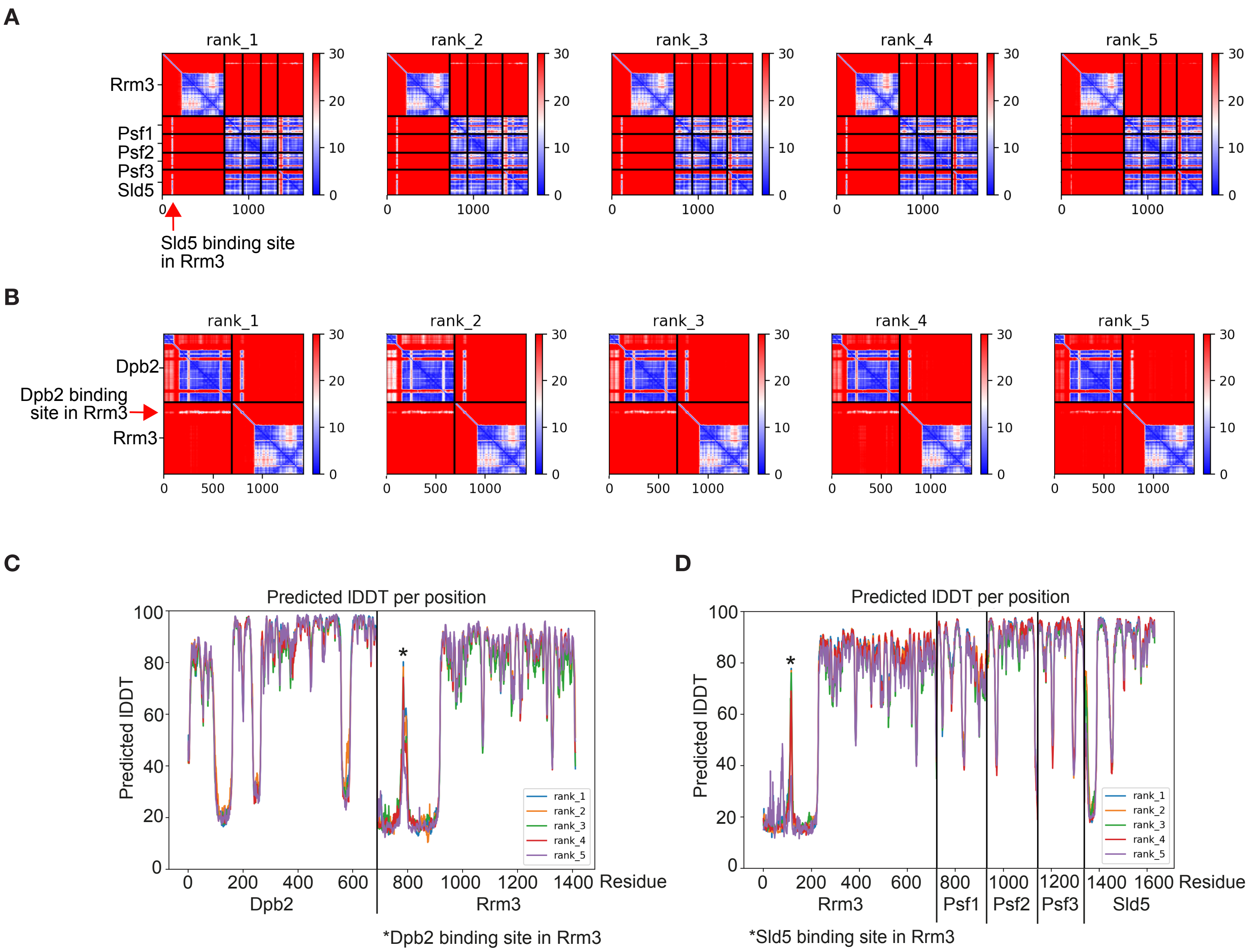

Appendix Figure S1

Supporting data for AlphaFold-Multimer modelling of Rrm3 in the budding yeast replisome.

(**A-B**) Predicted Alignment Error (PAE) plots generated by the five AlphaFold-Multimer models for complexes of Rrm3 with (**A**) the tetrameric GINS complex and (**B**) Dpb2. The Short Linear Interaction Motifs (SLIMs) in the Rrm3 IDR that are predicted to interact with Sld5 and Dpb2 are indicated with red arrowheads. PAE values are in Angstroms. (**C-D**) pLDDT values for Rrm3 in complex with Dpb2 (**C**) and GINS (**D**) displayed as graphs. pLDDT values for each of the five AlphaFold-Multimer models are shown as differently coloured lines. Binding of Dpb2 and Sld5 to disordered regions within the Rrm3 IDR induce large increases in pLDDT values at the predicted binding interface, as indicated by *.

Olson et al, Appendix Figure S2

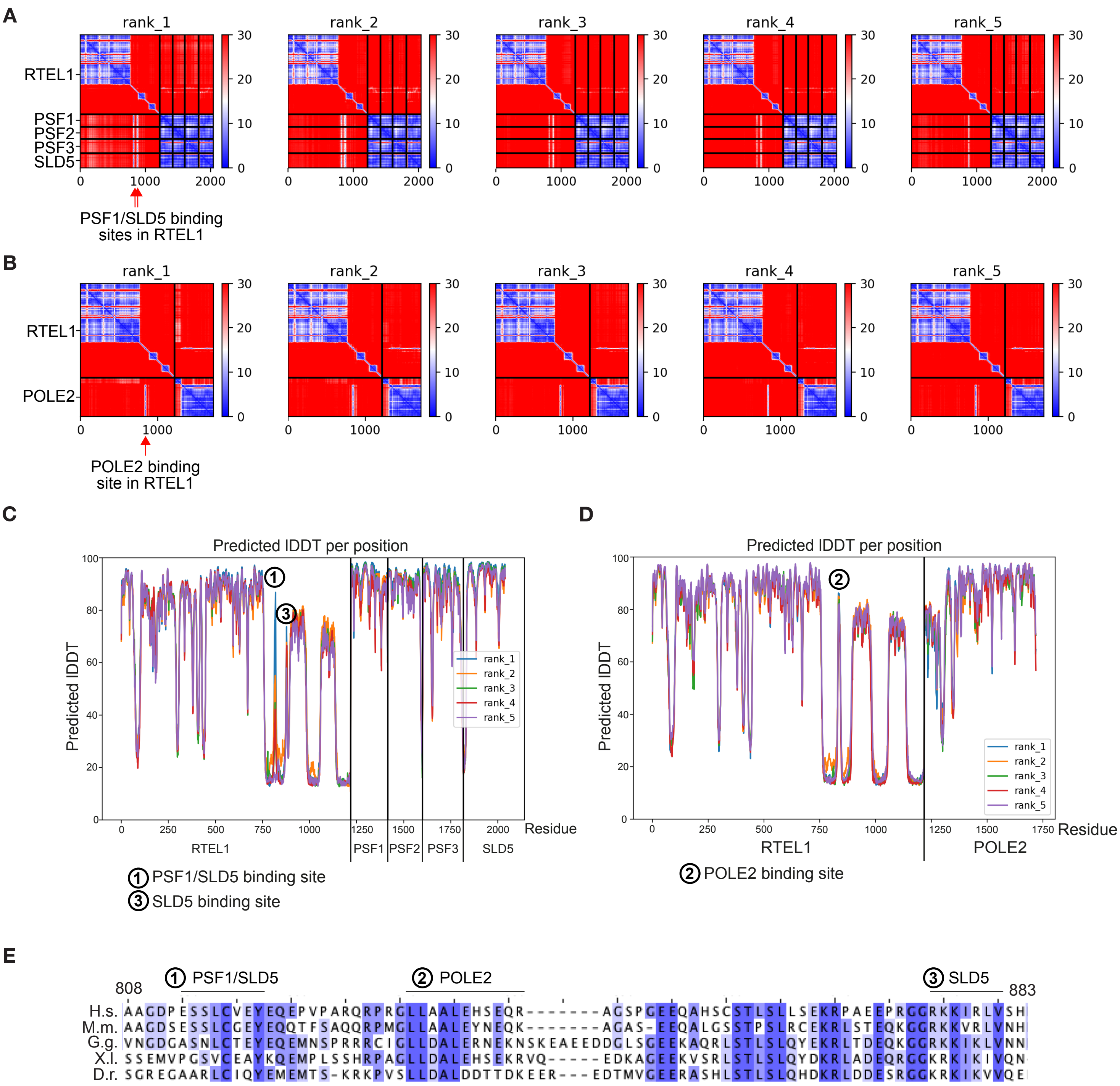

Appendix Figure S2

Supporting data for AlphaFold-Multimer modelling of RTEL1 in the human replisome. (**A-B**) Predicted Alignment Error (PAE) plots generated by the five AlphaFold-Multimer models for complexes of RTEL1 with (**A**) the tetrameric GINS complex and (**B**) POLE2. The Short Linear Interaction Motifs (SLIMs) in RTEL1 that are predicted to interact with SLD5 / PSF1 and POLE2 are indicated with red arrowheads. PAE values are in Angstroms. (**C-D**) pLDDT values for RTEL1 in complex with GINS (**C**) and POLE2 (**D**) displayed as graphs. pLDDT values for each of the five AlphaFold-Multimer models are shown as differently coloured lines. Binding of POLE2 and GINS to disordered regions within RTEL1 induce large increases in pLDDT values at the predicted binding interface, as indicated by the numbers 1-3, which correspond to the numbering of the binding sites in Fig 6. (**E**) RTEL1 sequence alignments generated in Jalview using Clustal, coloured according to percentage identity. RTEL1 from various metazoan species is included (H.s., *Homo sapiens*, M.m. *Mus musculus*, G.g. *Gallus gallus*, X.l. *Xenopus laevis*, D.r. *Danio rerio*). Residue numbers correspond to *H. sapiens* RTEL1. CMGE-binding sites are labelled and numbered as in (**D**) and Fig 6.
