# Supplementary figures and images for: A common mechanism for recruiting the Rrm3 and RTEL1 accessory helicases to the eukaryotic replisome
Source: EMBO J. 2024 Jul 22;43(18):3. doi: 10.1038/s44318-024-00168-4 (PMC11405395; doi:10.1038/s44318-024-00168-4)

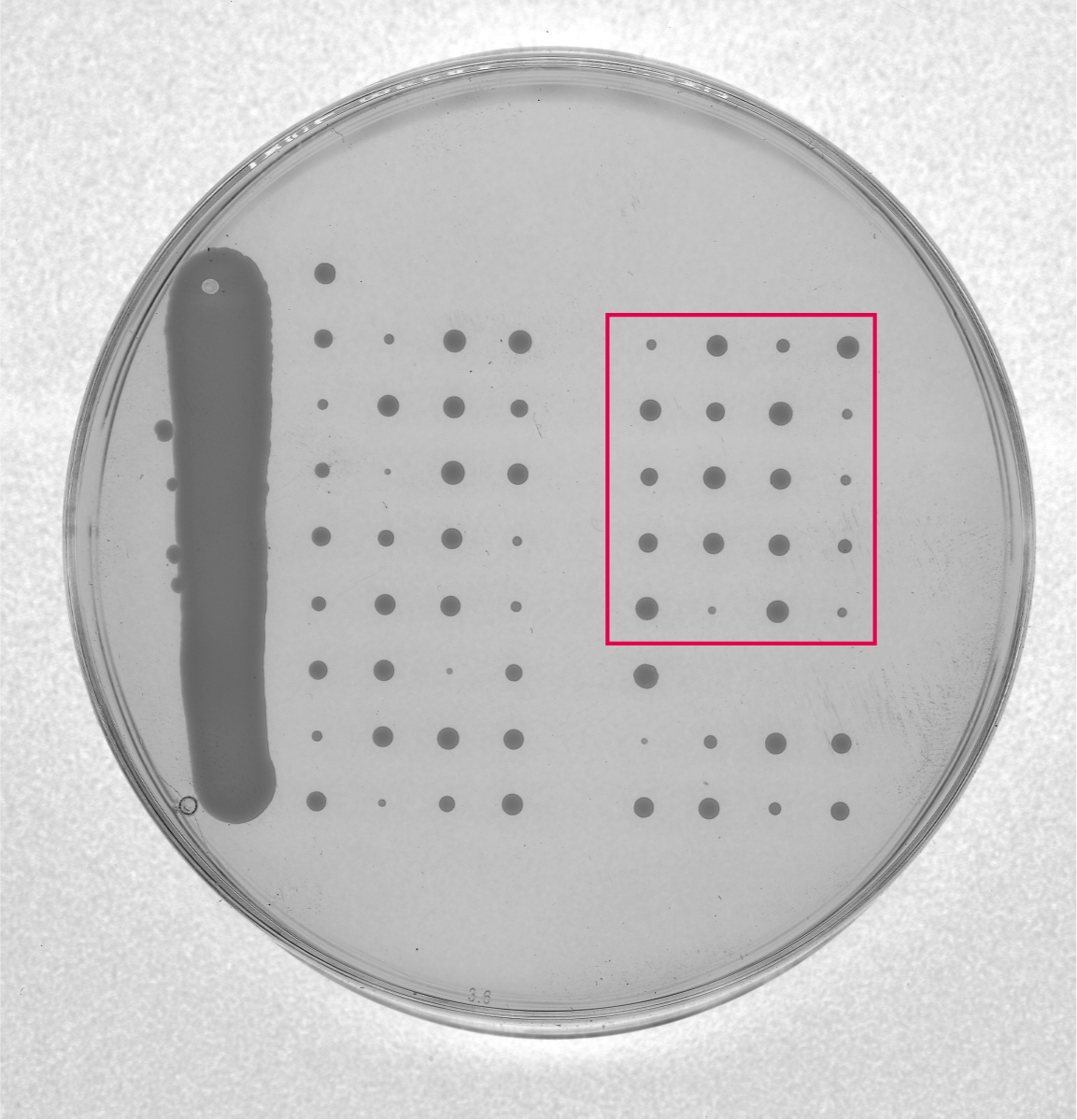

Supplement: Supplementary file 3 — Source data Fig. 1 [file 44318_2024_168_MOESM3_ESM.zip › Figure 1/1E/1E.tif]

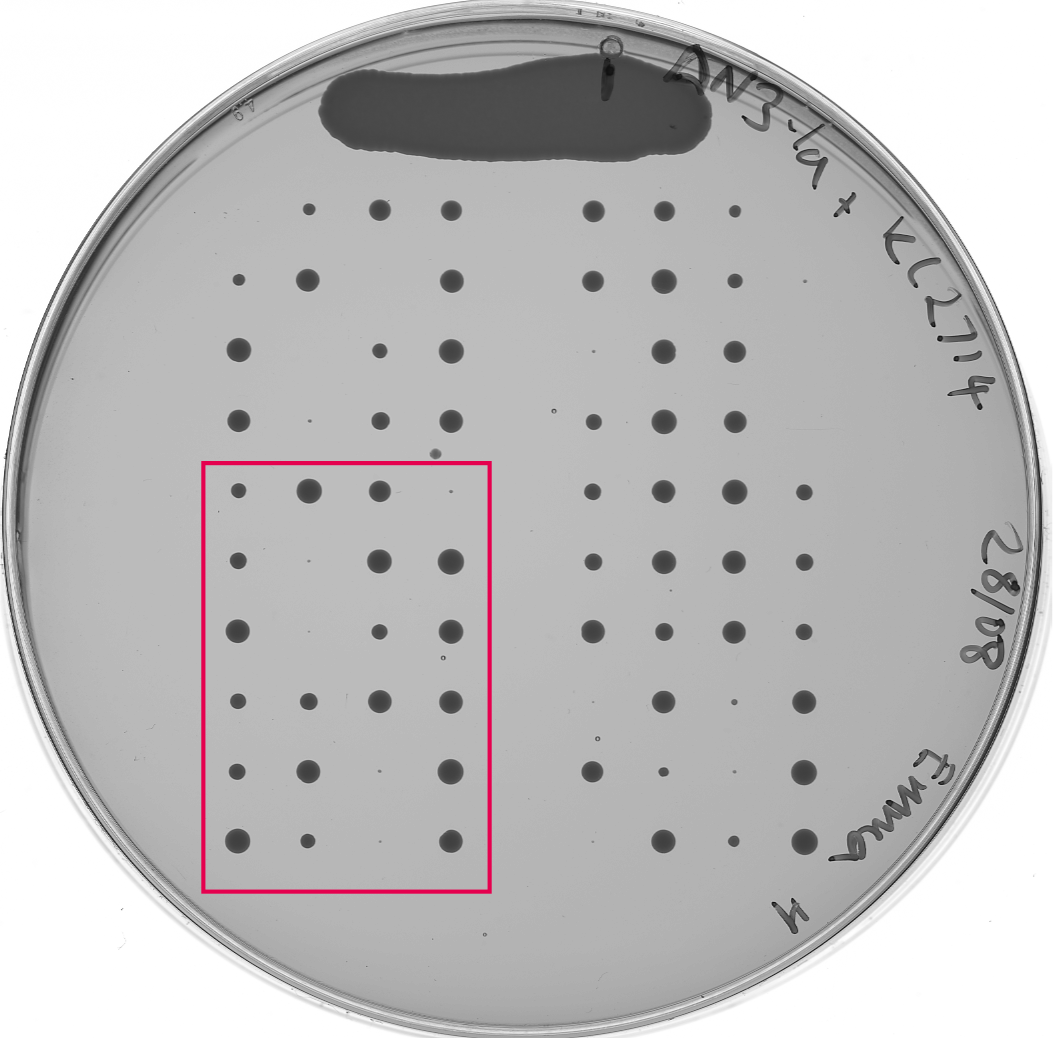

Supplement: Supplementary file 3 — Source data Fig. 1 [file 44318_2024_168_MOESM3_ESM.zip › Figure 1/1D/1D.tif]

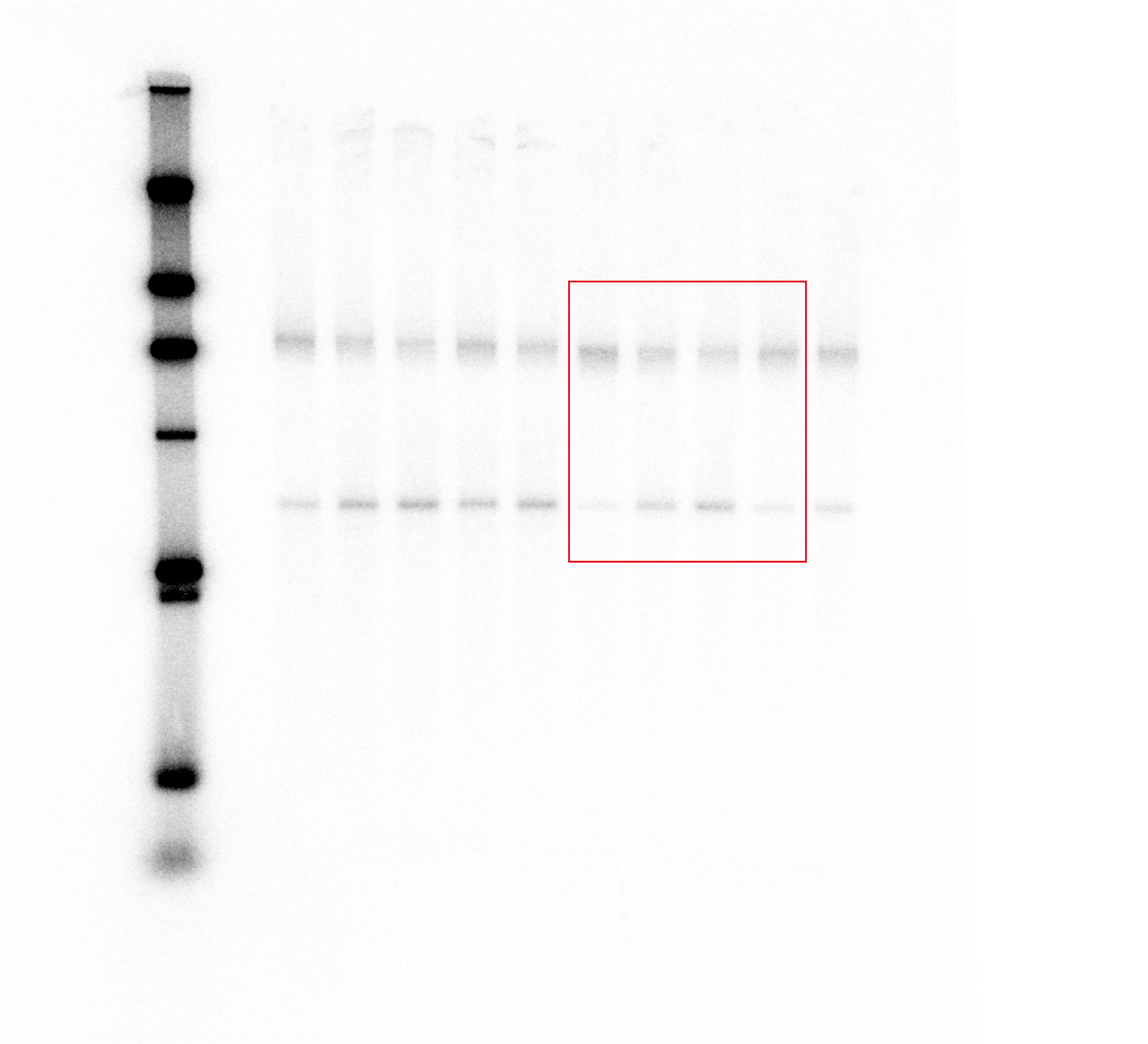

Supplement: Supplementary file 3 — Source data Fig. 1 [file 44318_2024_168_MOESM3_ESM.zip › Figure 1/1C/1C.tif]

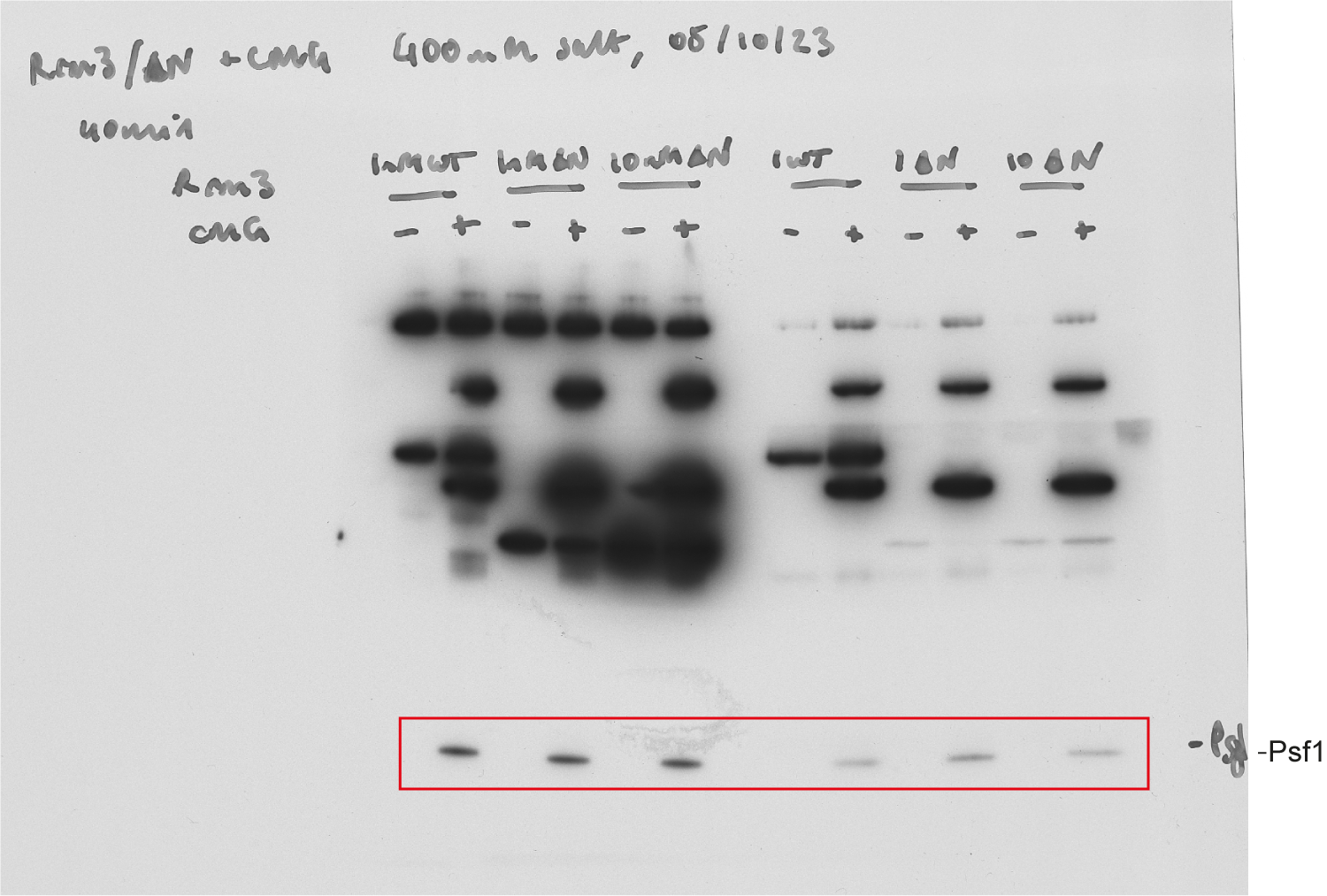

Supplement: Supplementary file 4 — Source data Fig. 2 [file 44318_2024_168_MOESM4_ESM.zip › Figure 2/2A/2A_Psf1.tif]

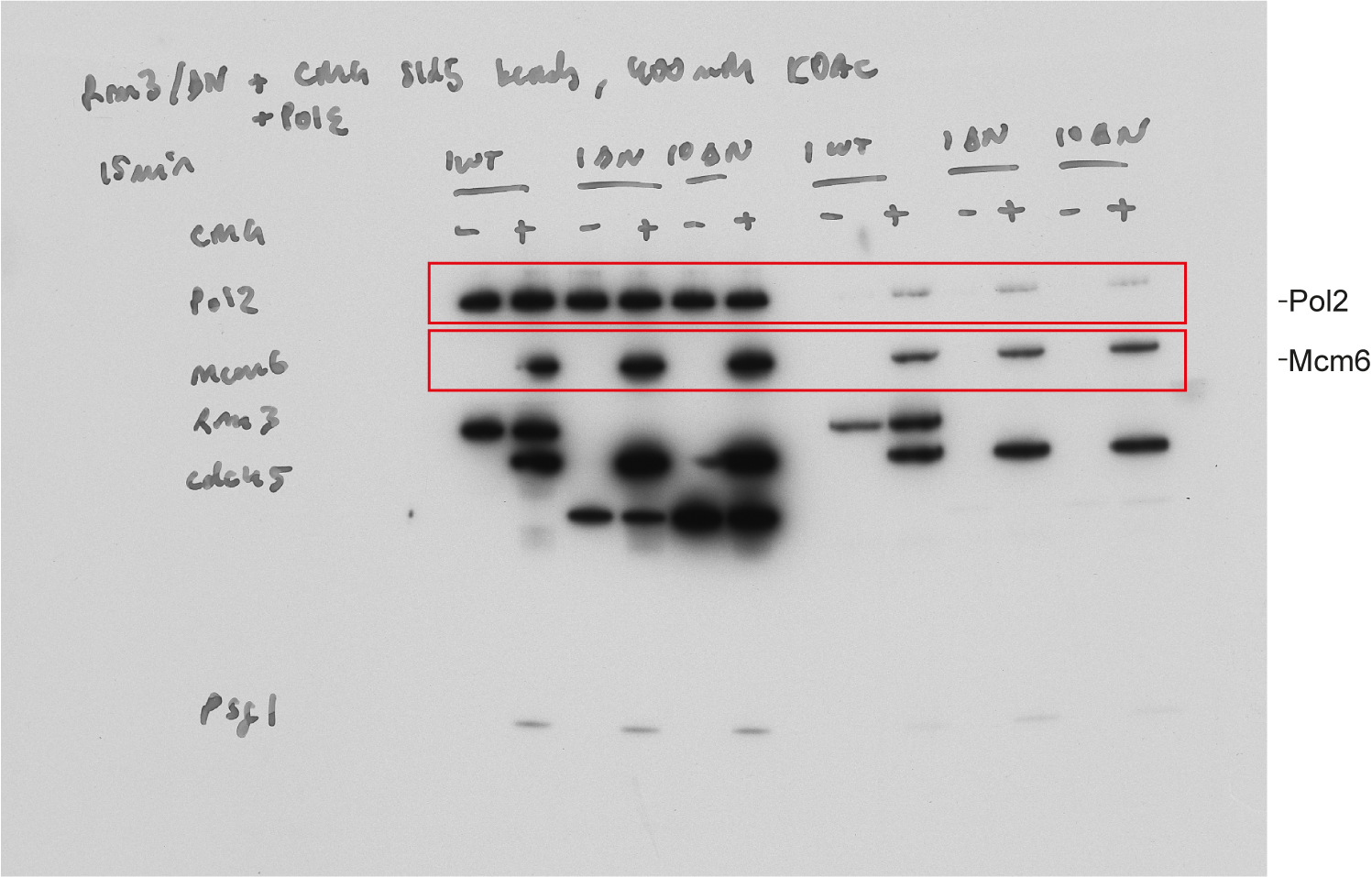

Supplement: Supplementary file 4 — Source data Fig. 2 [file 44318_2024_168_MOESM4_ESM.zip › Figure 2/2A/2A_Pol2_Mcm6.tif]

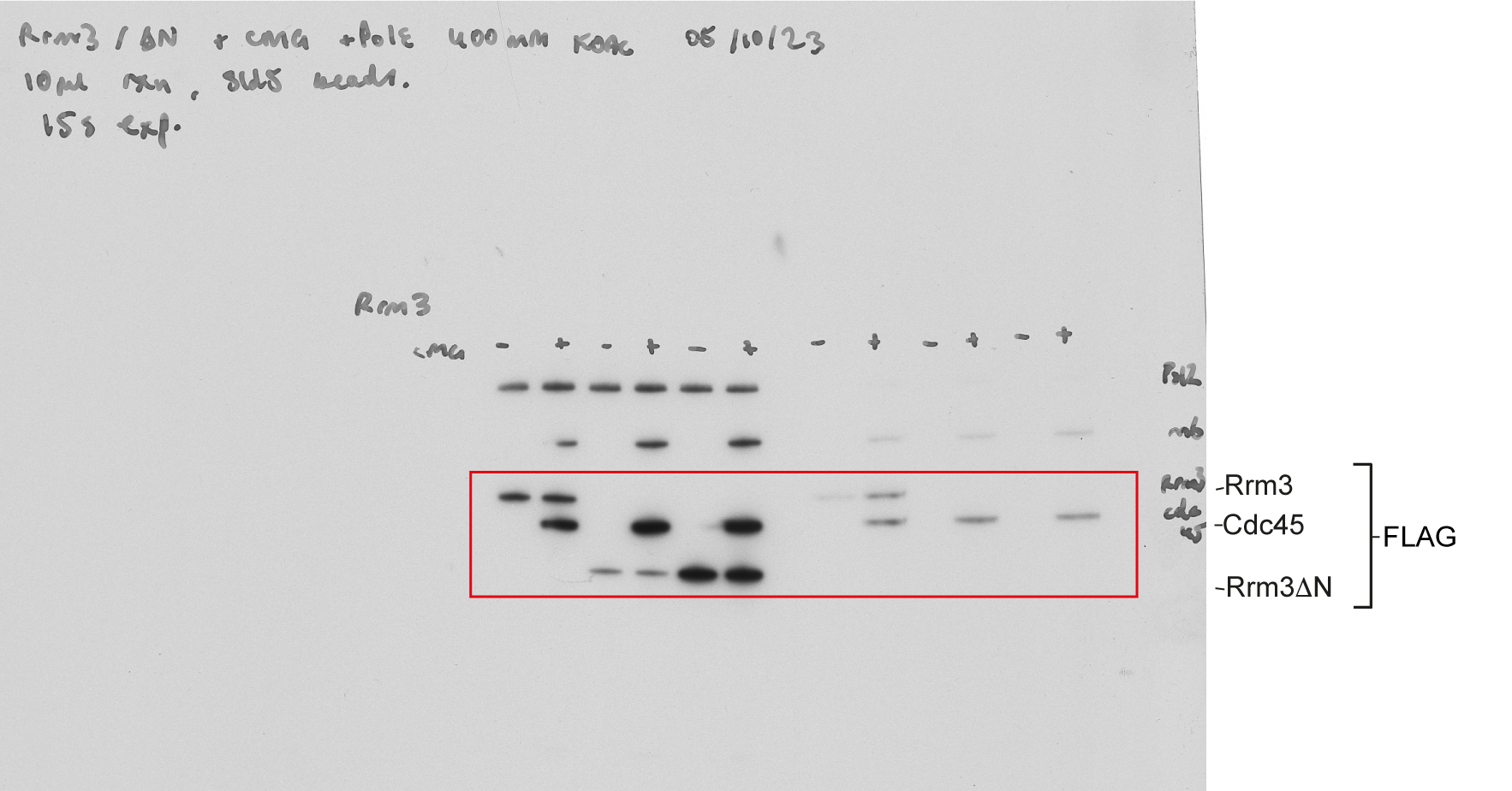

Supplement: Supplementary file 4 — Source data Fig. 2 [file 44318_2024_168_MOESM4_ESM.zip › Figure 2/2A/2A_FLAG.tif]

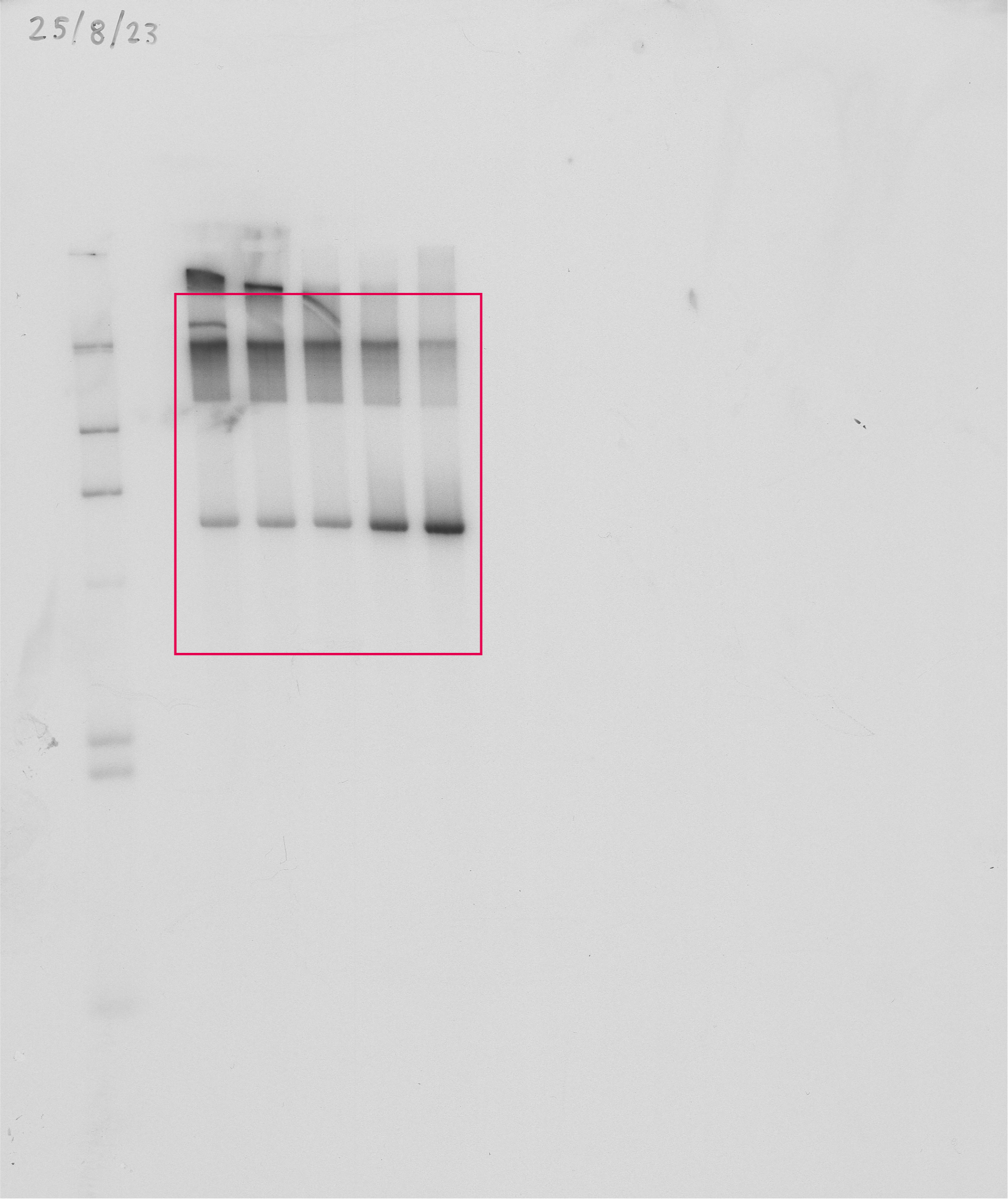

Supplement: Supplementary file 4 — Source data Fig. 2 [file 44318_2024_168_MOESM4_ESM.zip › Figure 2/2F/2F.tif]

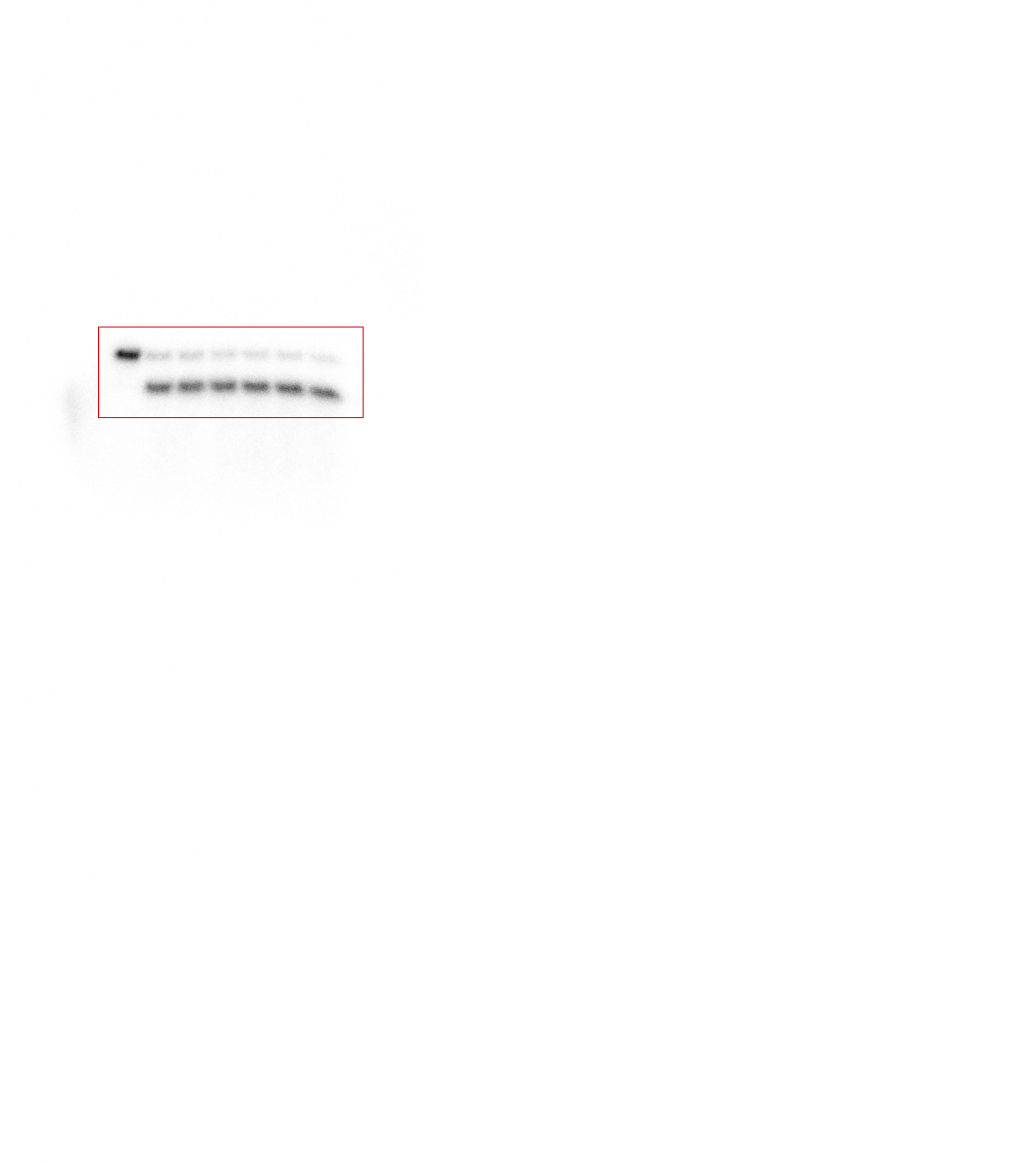

Supplement: Supplementary file 4 — Source data Fig. 2 [file 44318_2024_168_MOESM4_ESM.zip › Figure 2/2C/2C.tif]

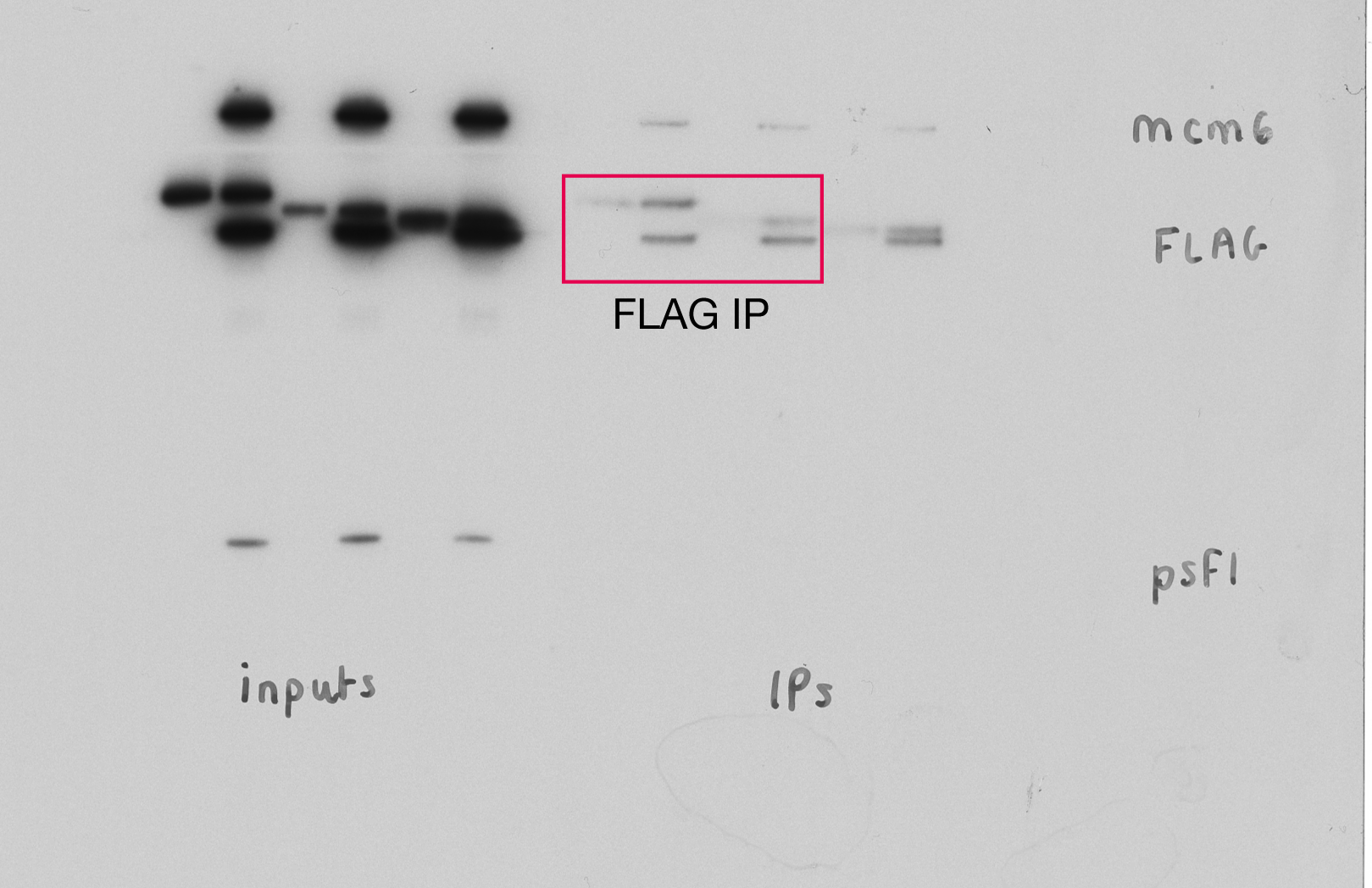

Supplement: Supplementary file 4 — Source data Fig. 2 [file 44318_2024_168_MOESM4_ESM.zip › Figure 2/2D/2D_flag.tif]

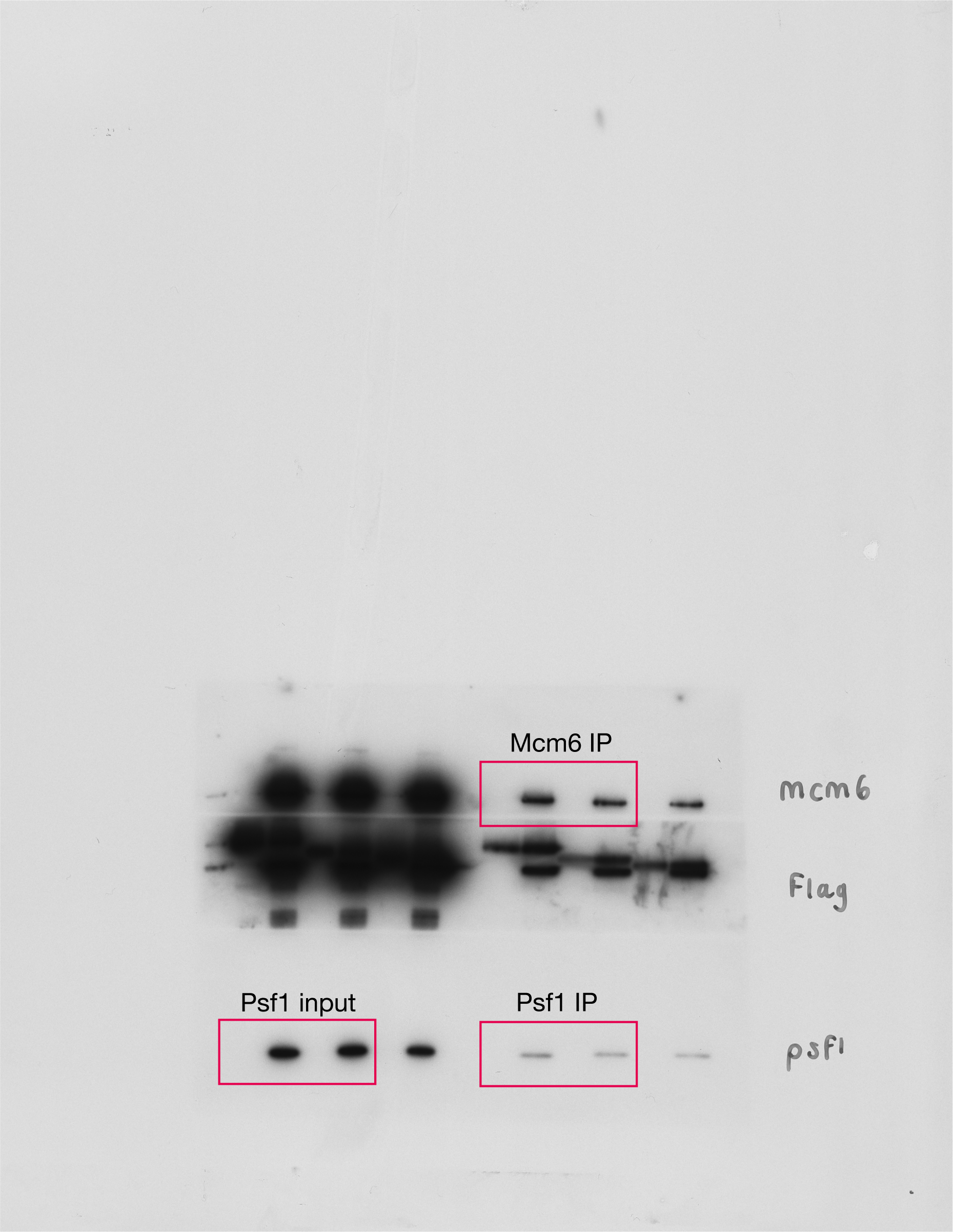

Supplement: Supplementary file 4 — Source data Fig. 2 [file 44318_2024_168_MOESM4_ESM.zip › Figure 2/2D/2D_psf1_mcm6.tif]

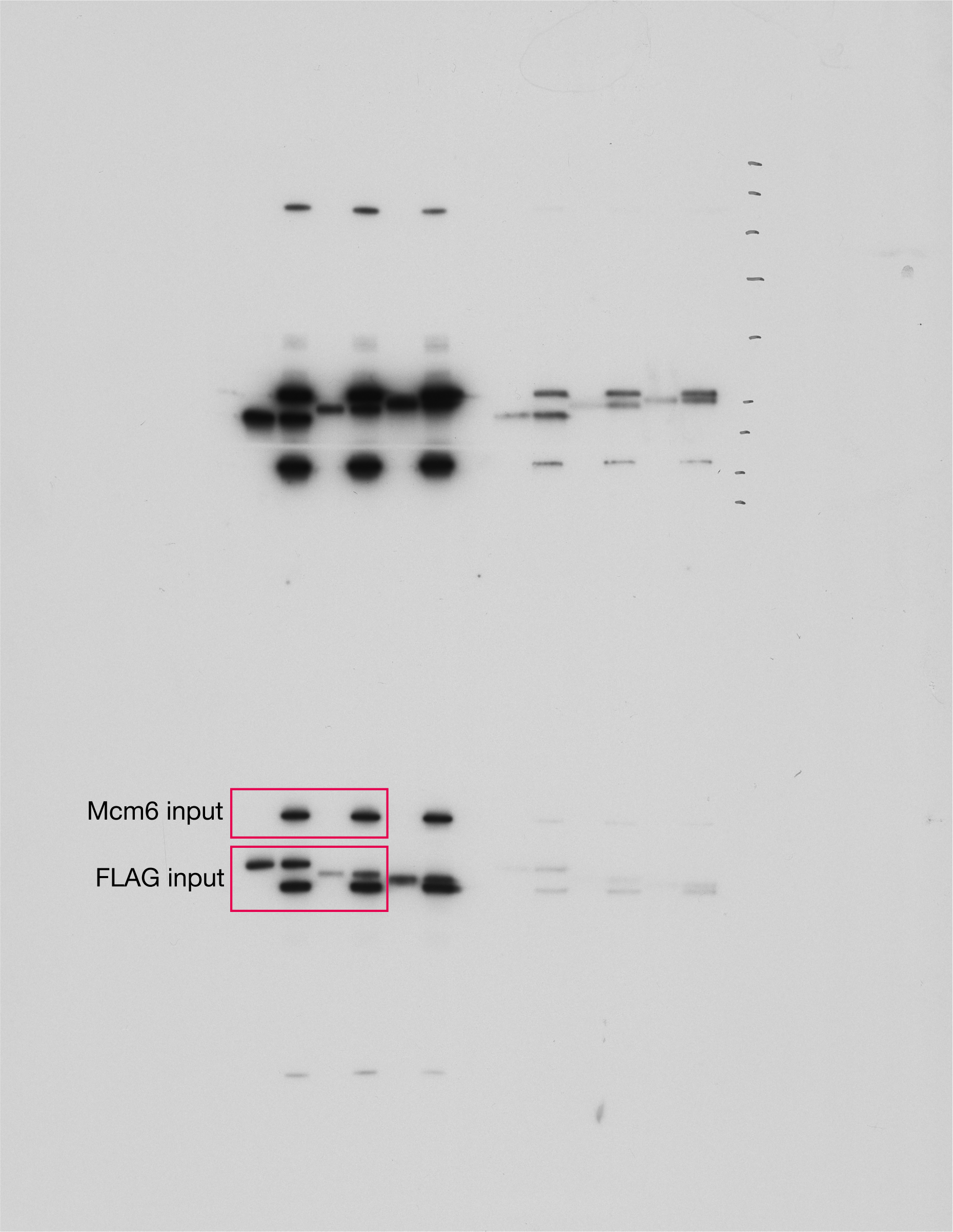

Supplement: Supplementary file 4 — Source data Fig. 2 [file 44318_2024_168_MOESM4_ESM.zip › Figure 2/2D/2D_flag_mcm6.tif]

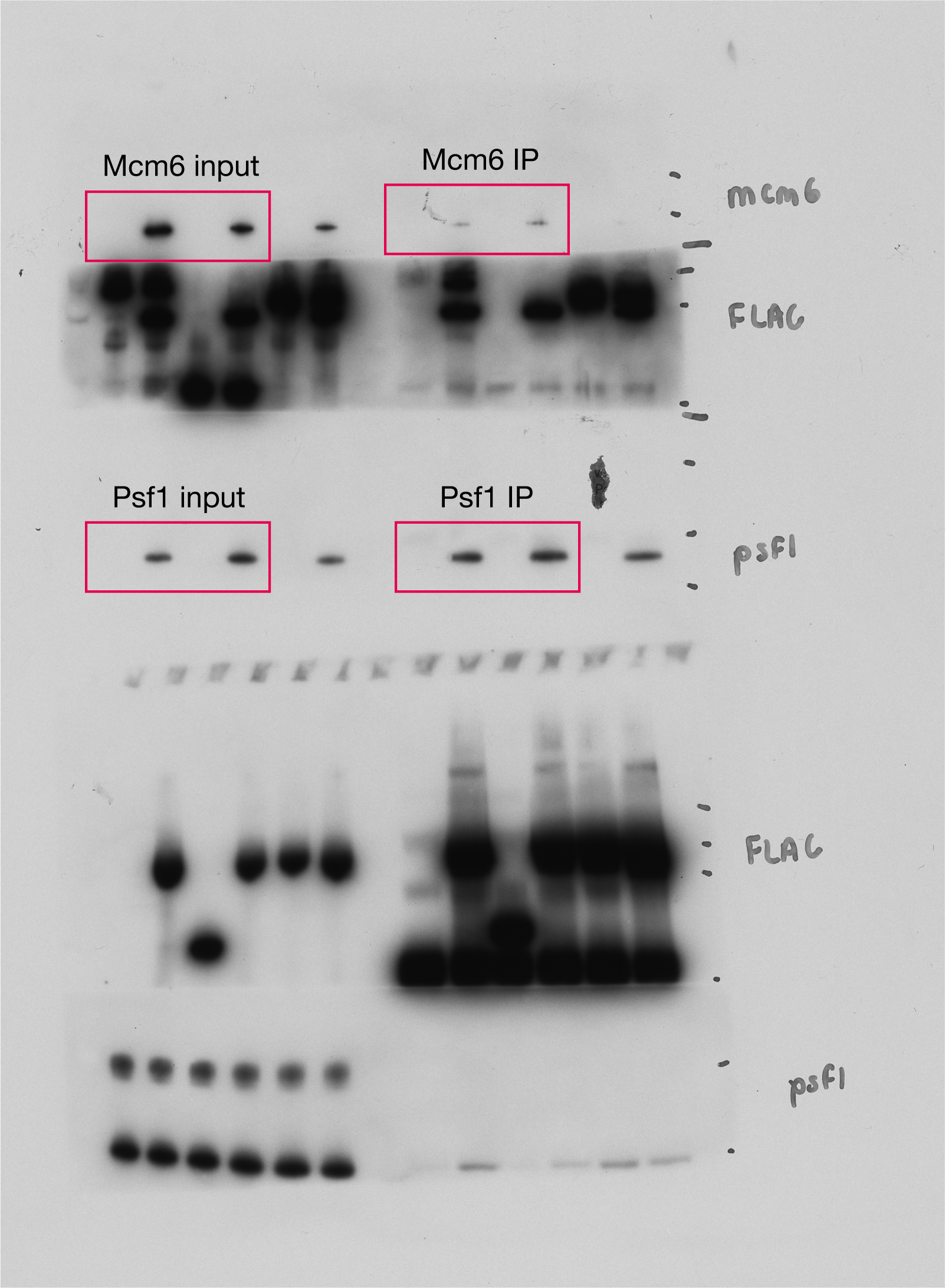

Supplement: Supplementary file 4 — Source data Fig. 2 [file 44318_2024_168_MOESM4_ESM.zip › Figure 2/2E/2E_Mcm6_Psf1.tif]

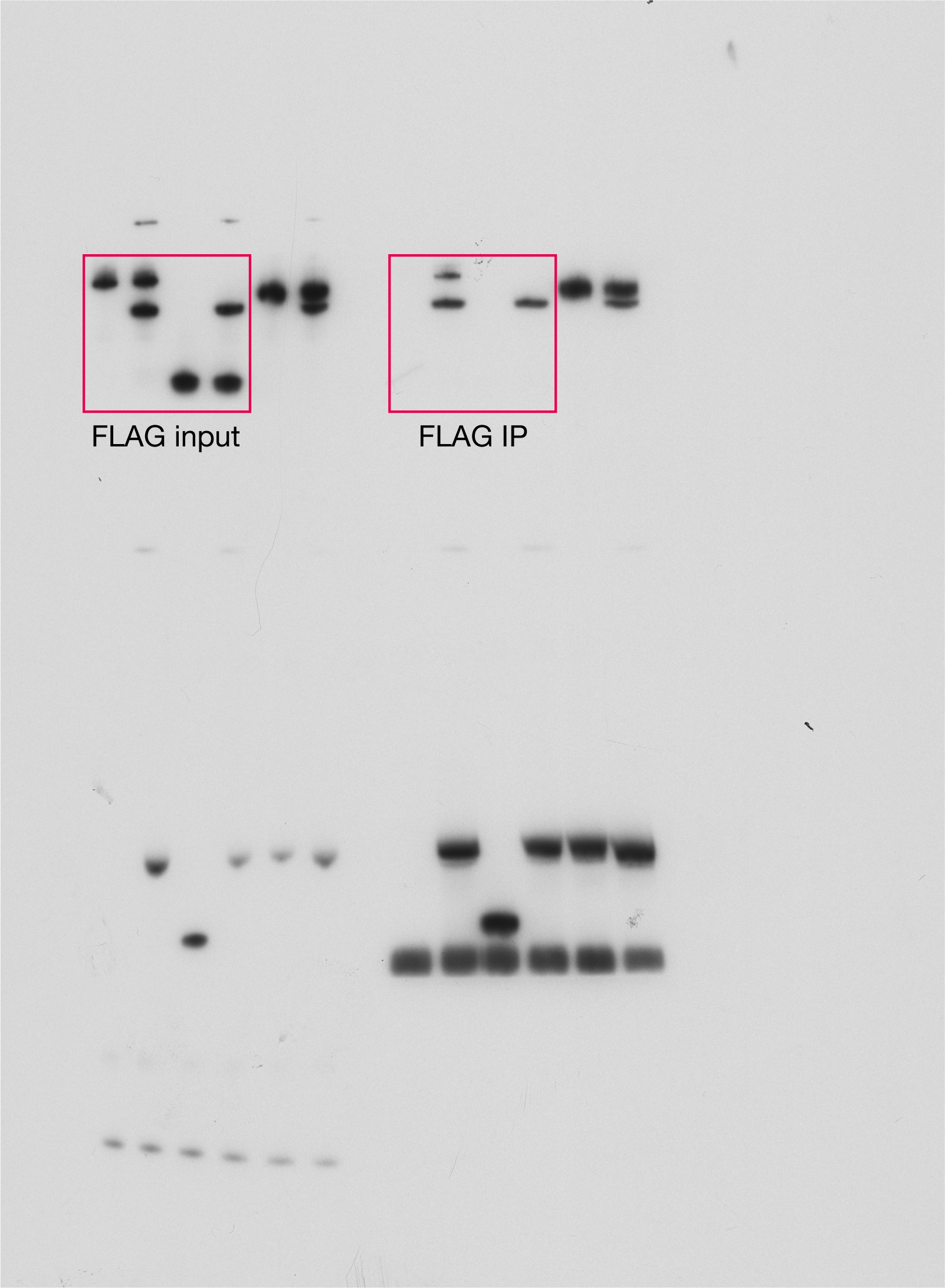

Supplement: Supplementary file 4 — Source data Fig. 2 [file 44318_2024_168_MOESM4_ESM.zip › Figure 2/2E/2E_FLAG.tif]

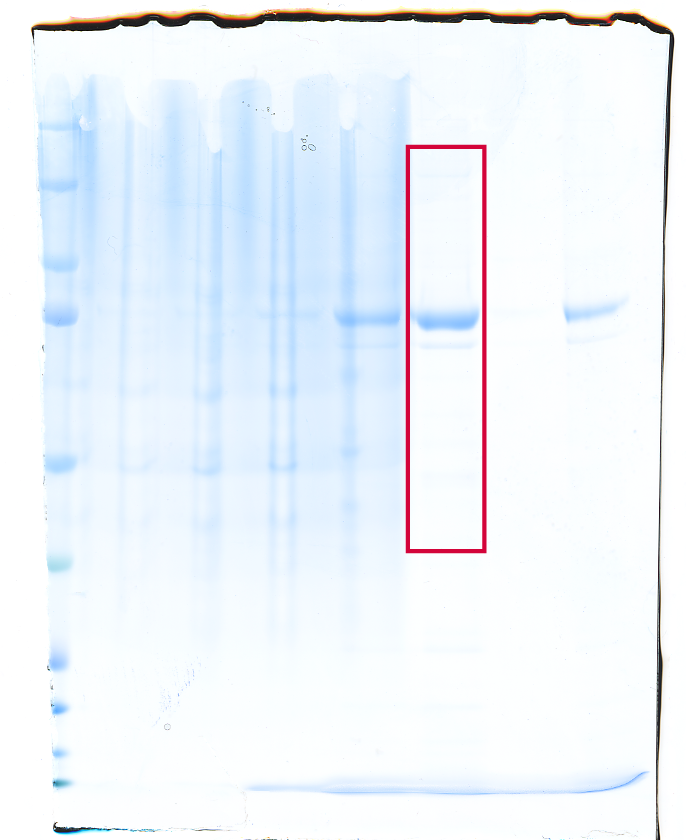

Supplement: Supplementary file 4 — Source data Fig. 2 [file 44318_2024_168_MOESM4_ESM.zip › Figure 2/2B/2B_Rrm3NBacPif1.tif]

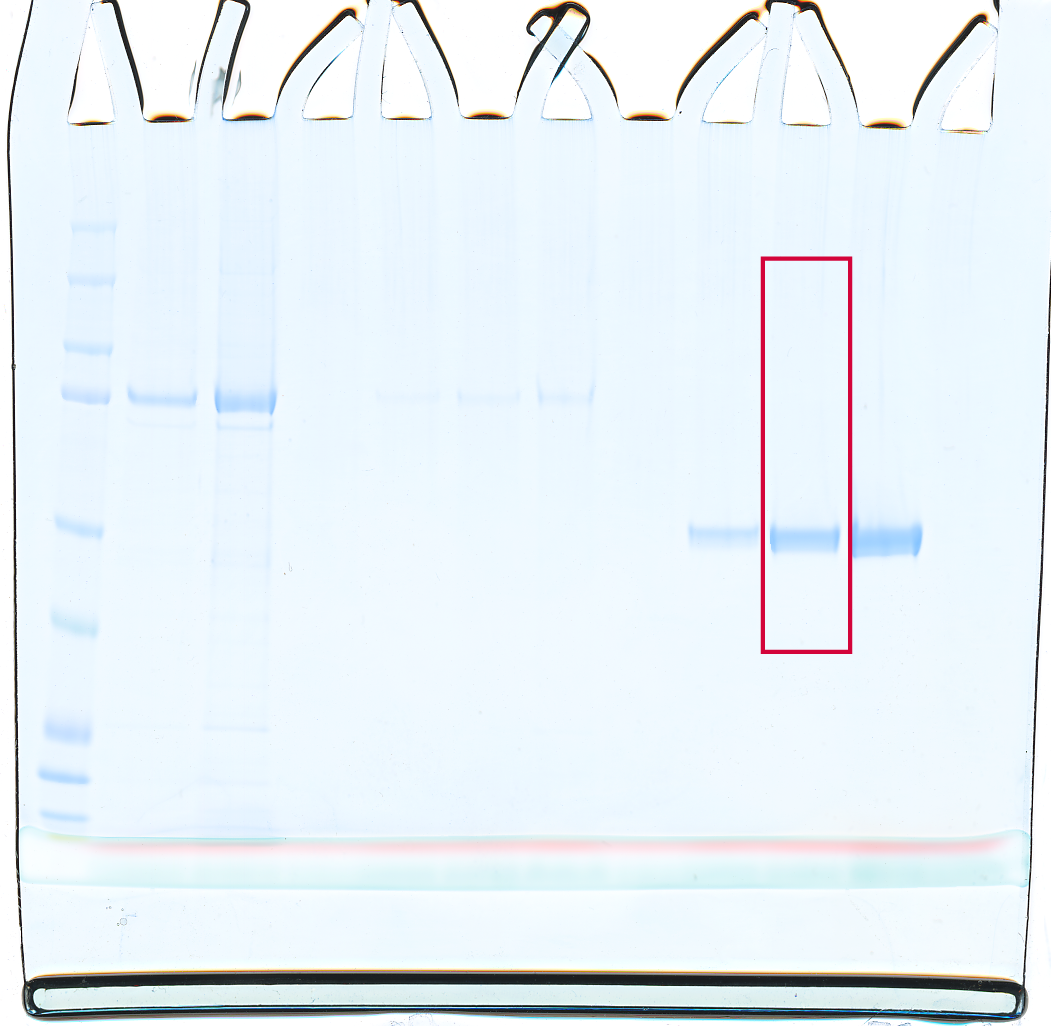

Supplement: Supplementary file 4 — Source data Fig. 2 [file 44318_2024_168_MOESM4_ESM.zip › Figure 2/2B/2B_BacPif1.tif]

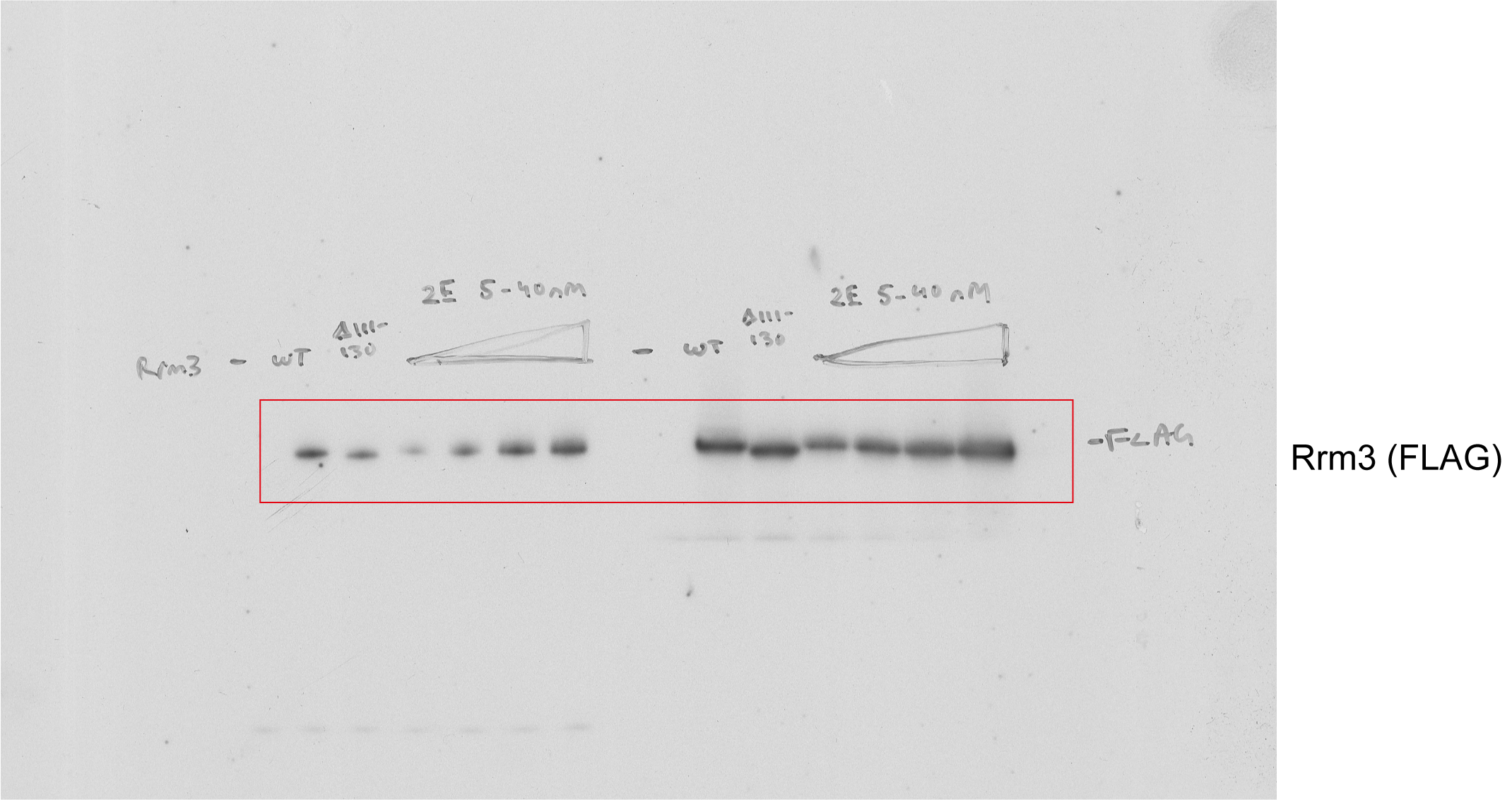

Supplement: Supplementary file 5 — Source data Fig. 4 [file 44318_2024_168_MOESM5_ESM.zip › Figure 4/4B/4B_Rrm3.tif]

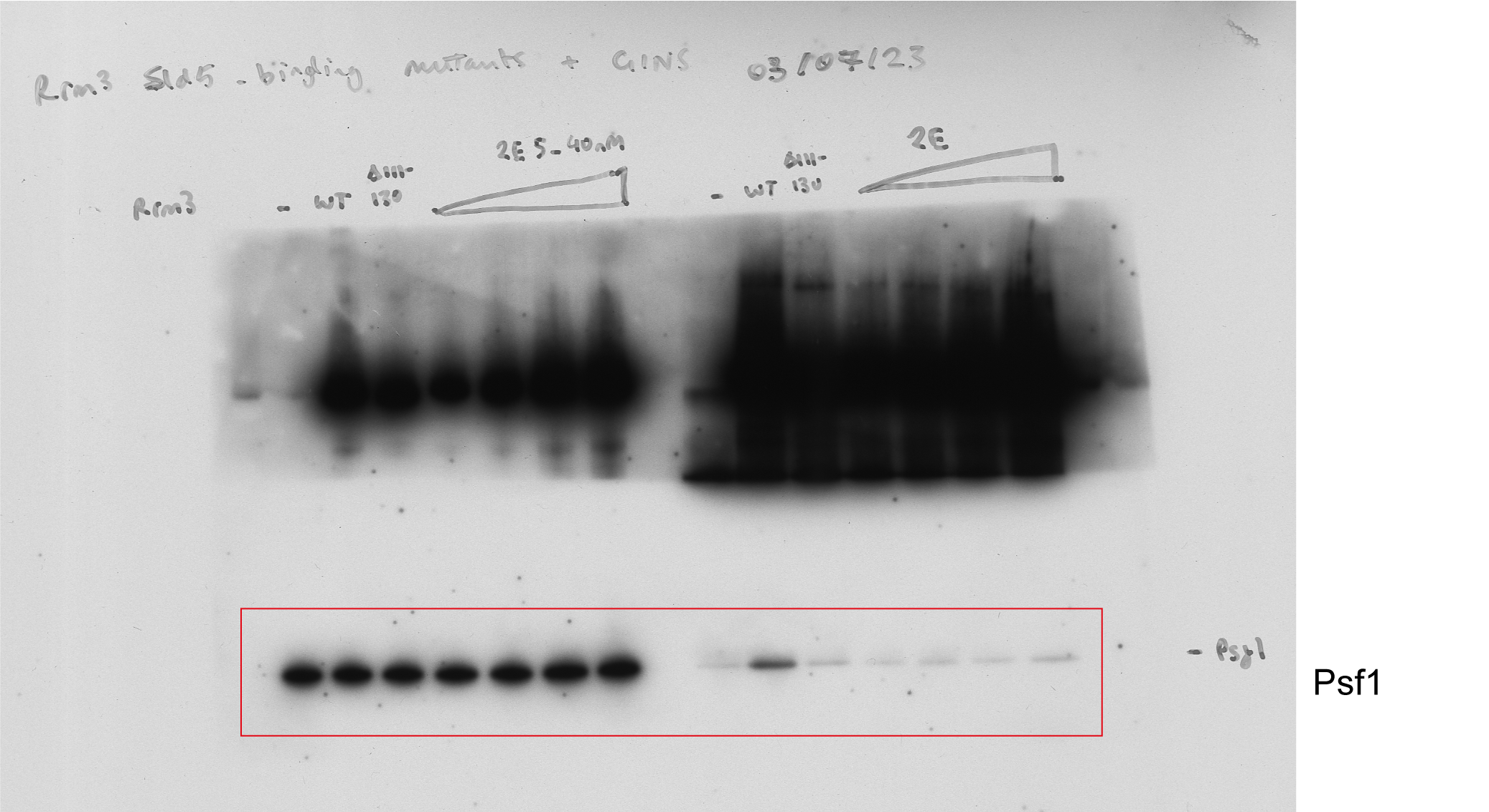

Supplement: Supplementary file 5 — Source data Fig. 4 [file 44318_2024_168_MOESM5_ESM.zip › Figure 4/4B/4B_Psf1.tif]

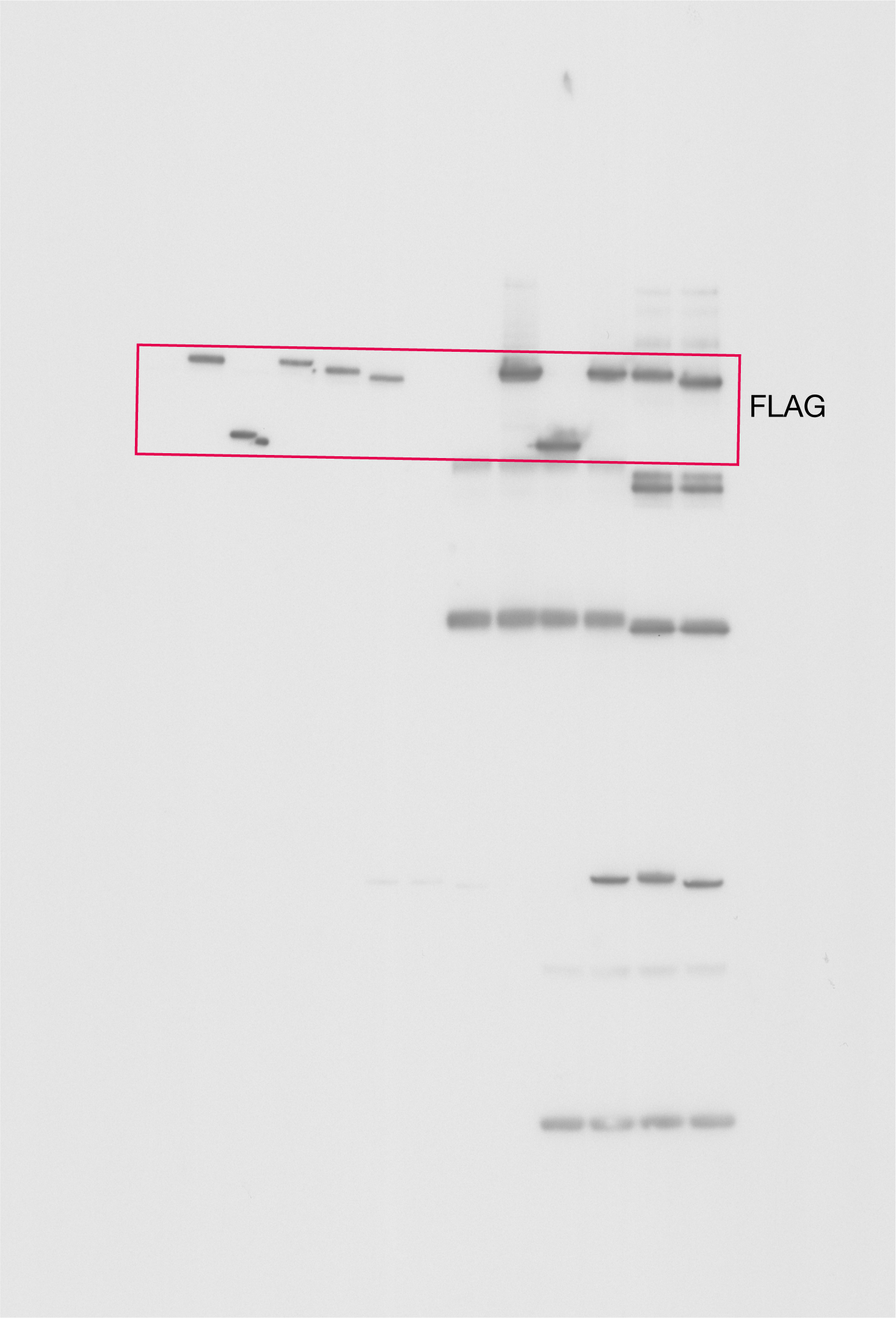

Supplement: Supplementary file 5 — Source data Fig. 4 [file 44318_2024_168_MOESM5_ESM.zip › Figure 4/4C/4C_FLAG.tif]

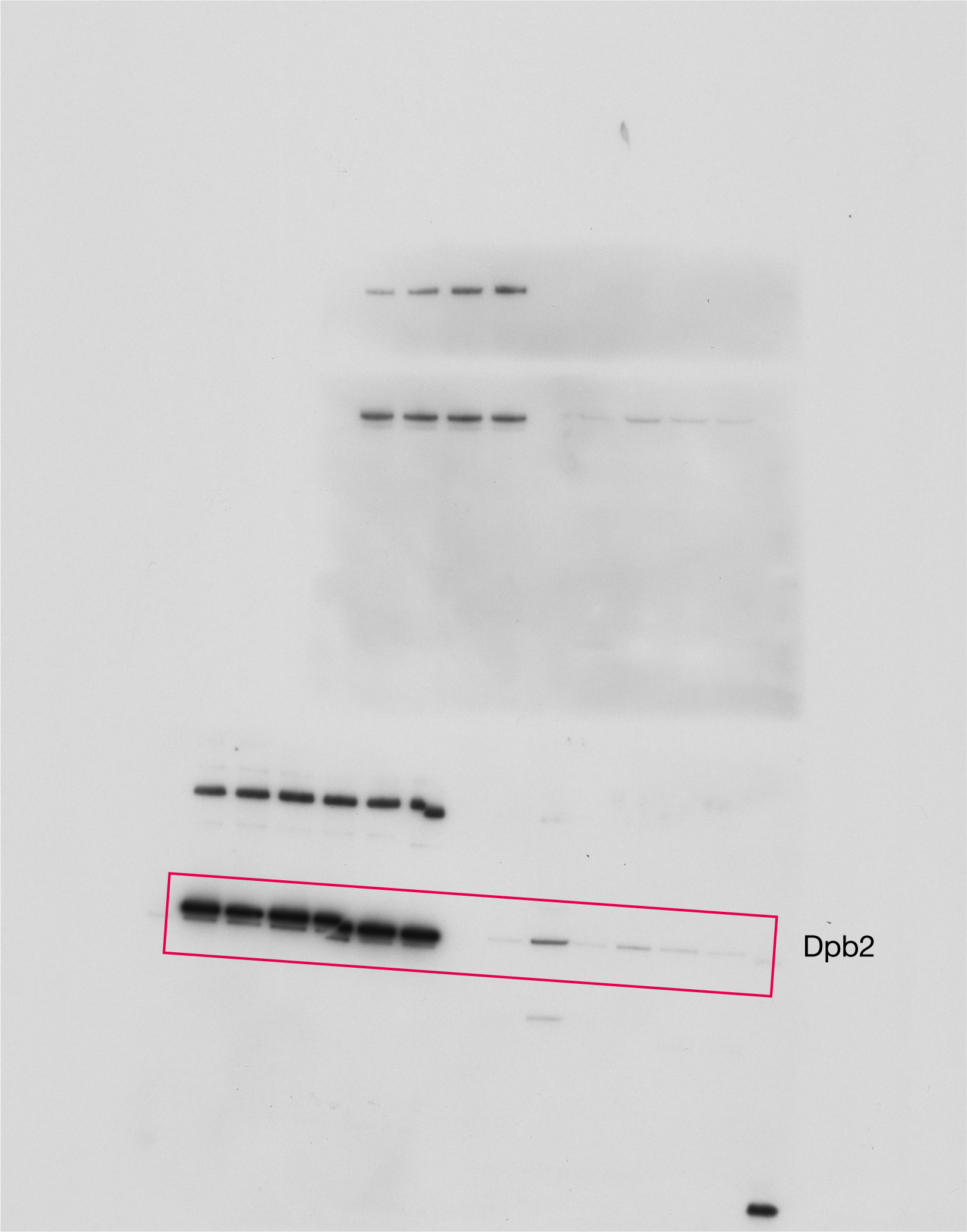

Supplement: Supplementary file 5 — Source data Fig. 4 [file 44318_2024_168_MOESM5_ESM.zip › Figure 4/4C/4C_dpb2.tif]

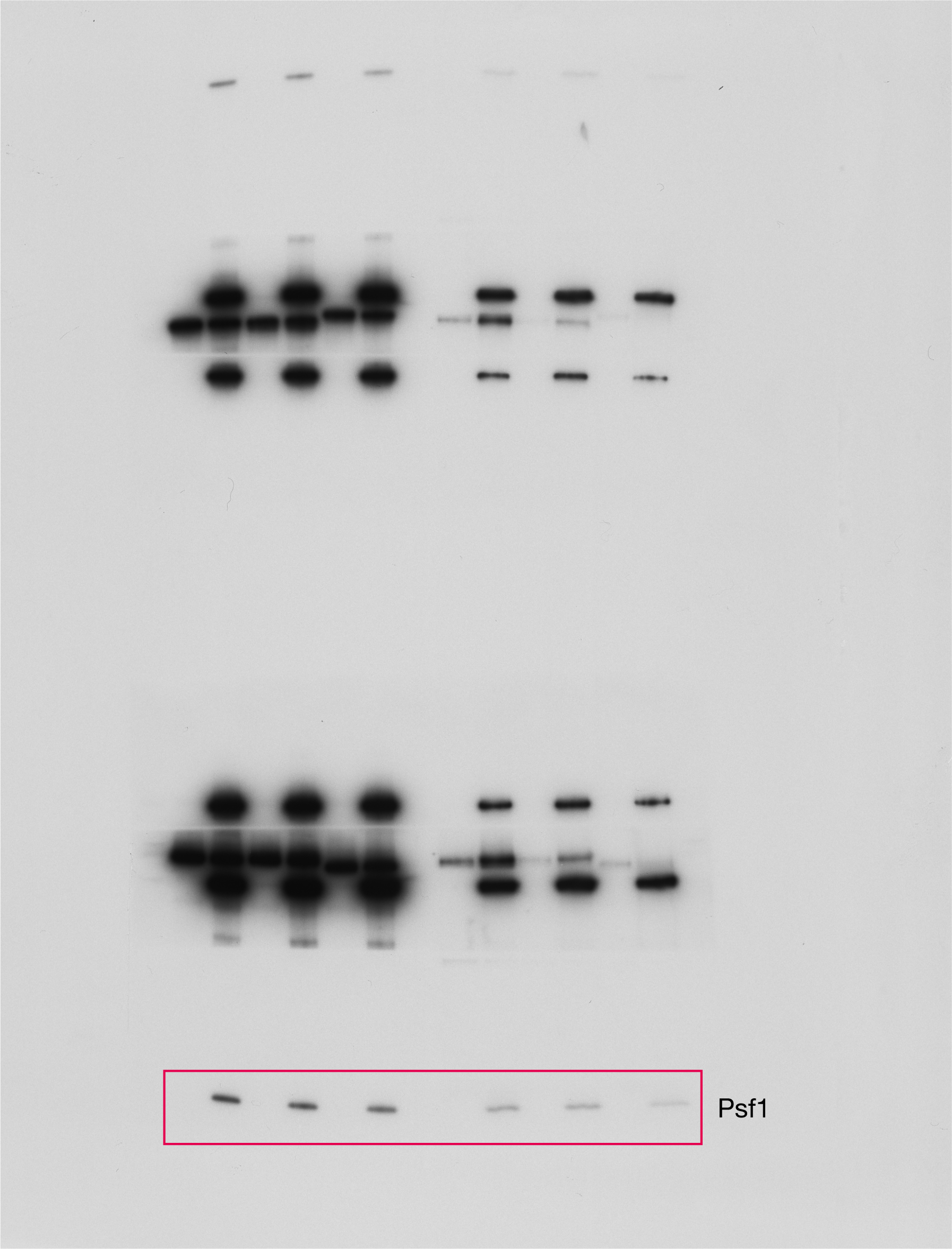

Supplement: Supplementary file 5 — Source data Fig. 4 [file 44318_2024_168_MOESM5_ESM.zip › Figure 4/4D/4D_Psf1.tif]

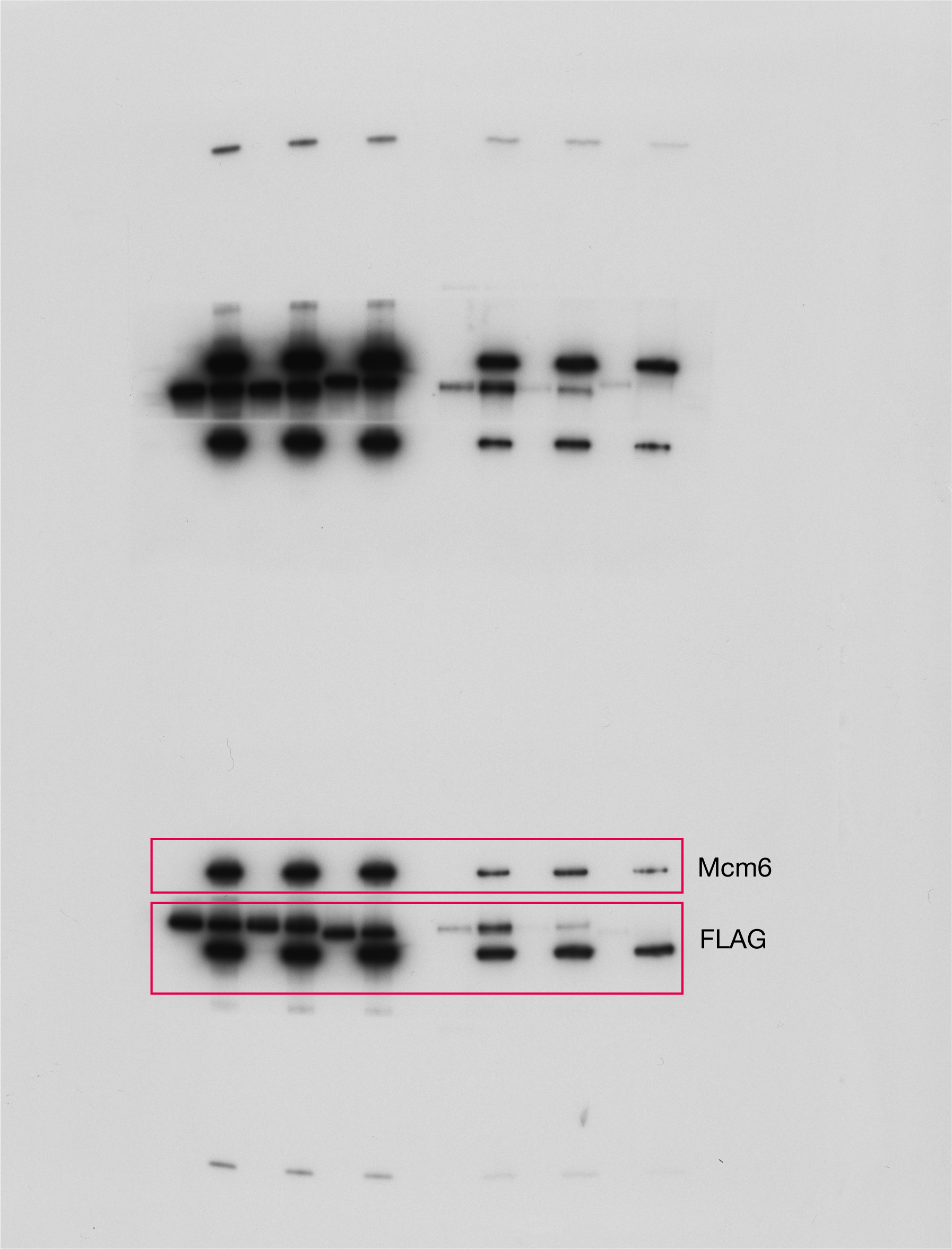

Supplement: Supplementary file 5 — Source data Fig. 4 [file 44318_2024_168_MOESM5_ESM.zip › Figure 4/4D/4D_FLAG_Mcm6.tif]

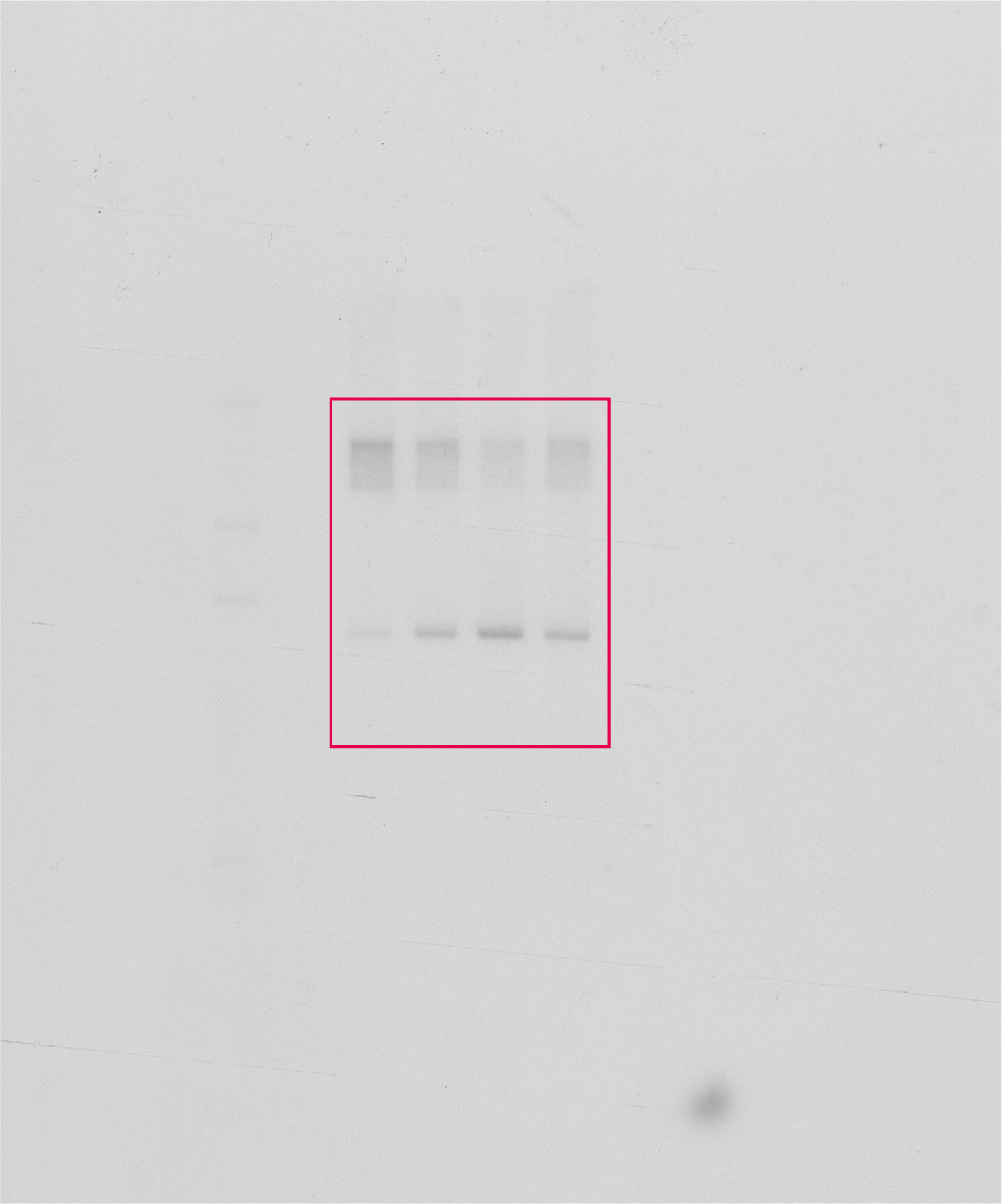

Supplement: Supplementary file 6 — Source data Fig. 5 [file 44318_2024_168_MOESM6_ESM.zip › Figure 5/5A/5A.tif]

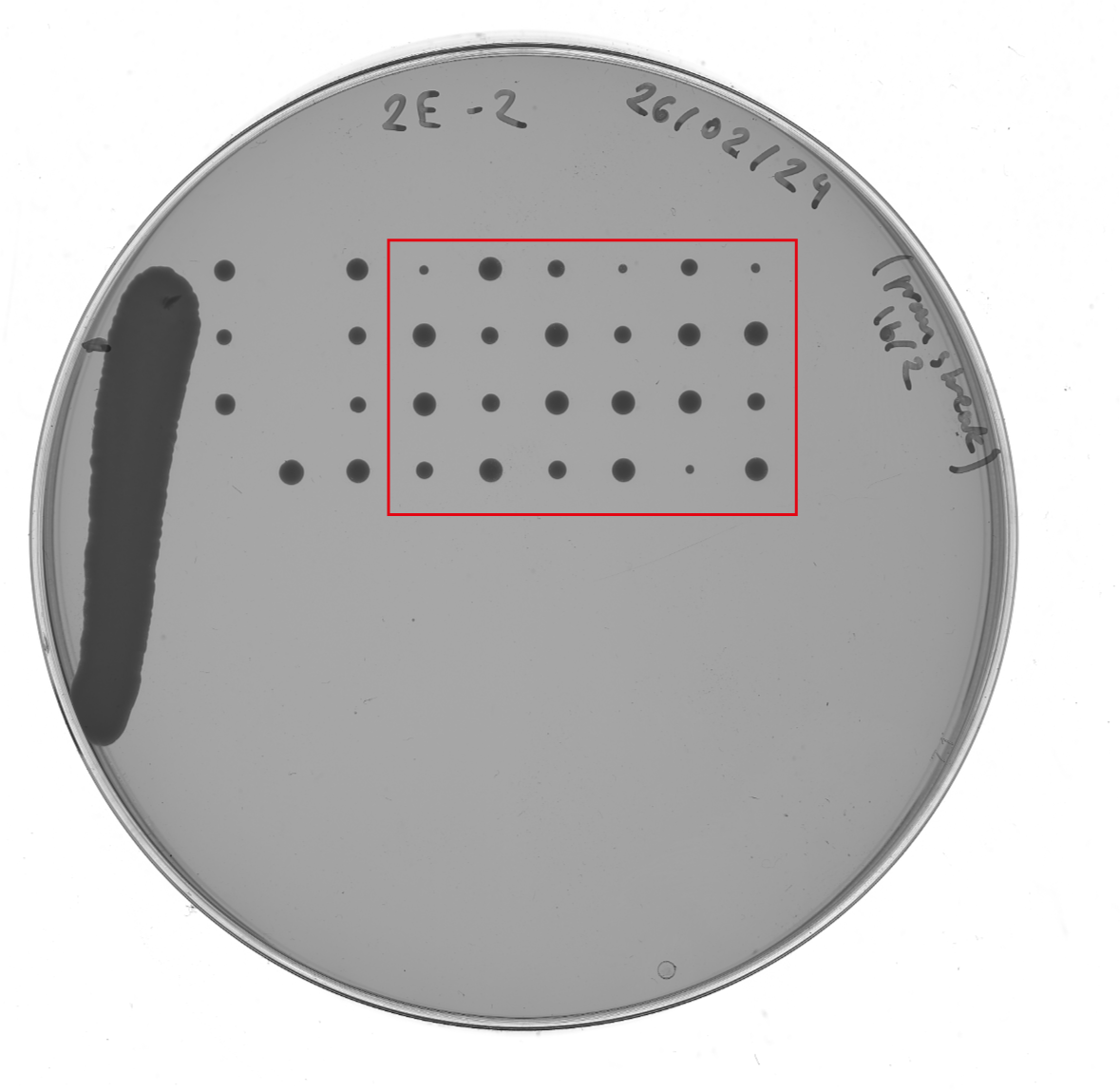

Supplement: Supplementary file 6 — Source data Fig. 5 [file 44318_2024_168_MOESM6_ESM.zip › Figure 5/5C/5C.tif]

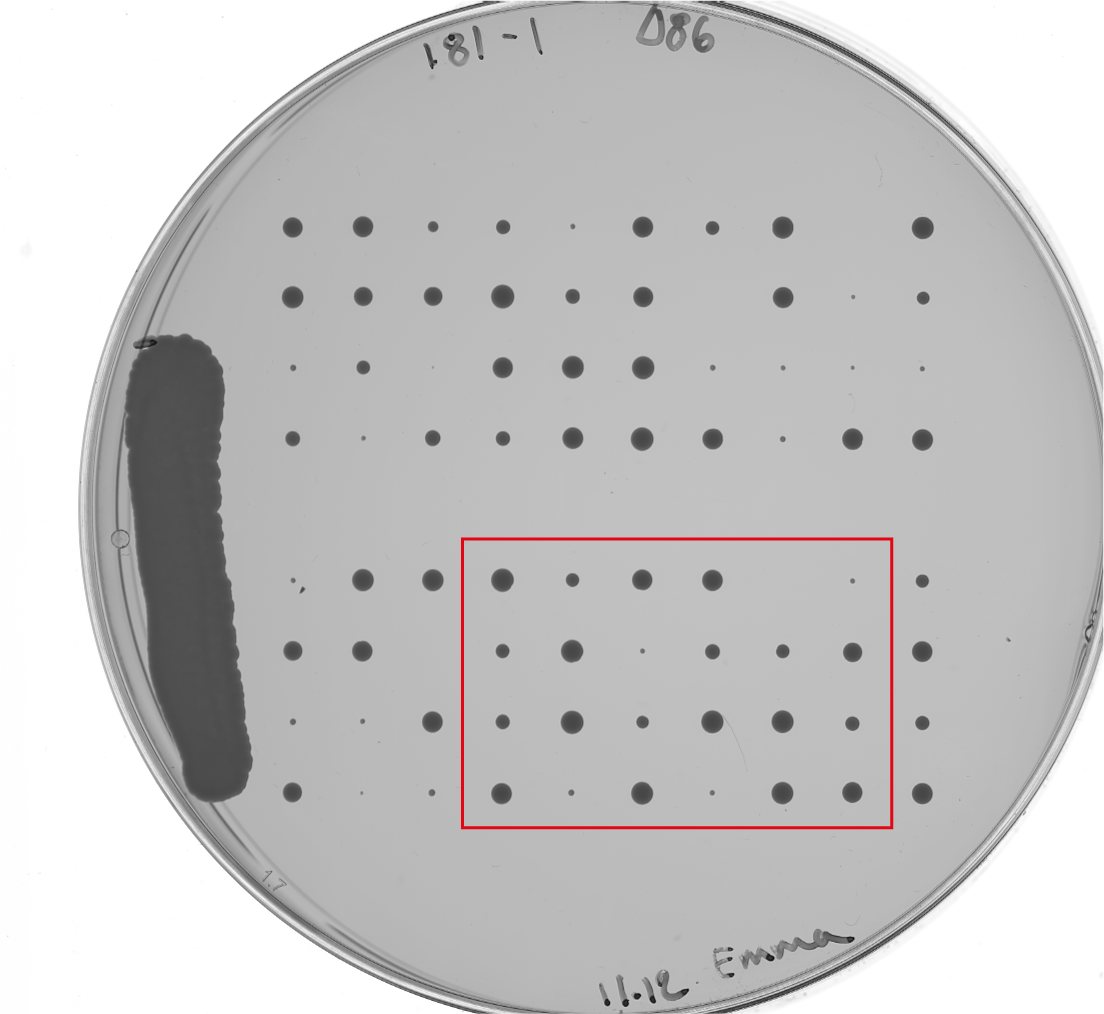

Supplement: Supplementary file 6 — Source data Fig. 5 [file 44318_2024_168_MOESM6_ESM.zip › Figure 5/5D/5D.tif]

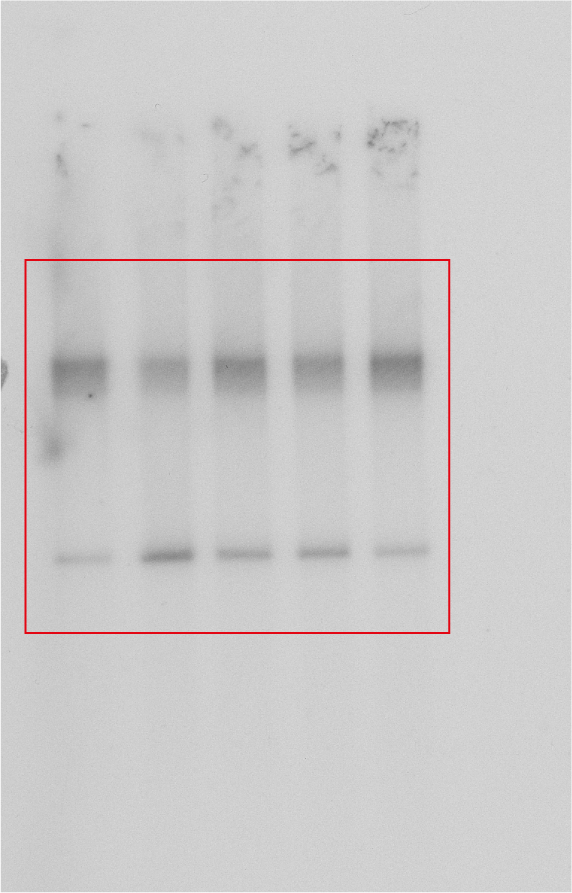

Supplement: Supplementary file 6 — Source data Fig. 5 [file 44318_2024_168_MOESM6_ESM.zip › Figure 5/5B/5B.tif]

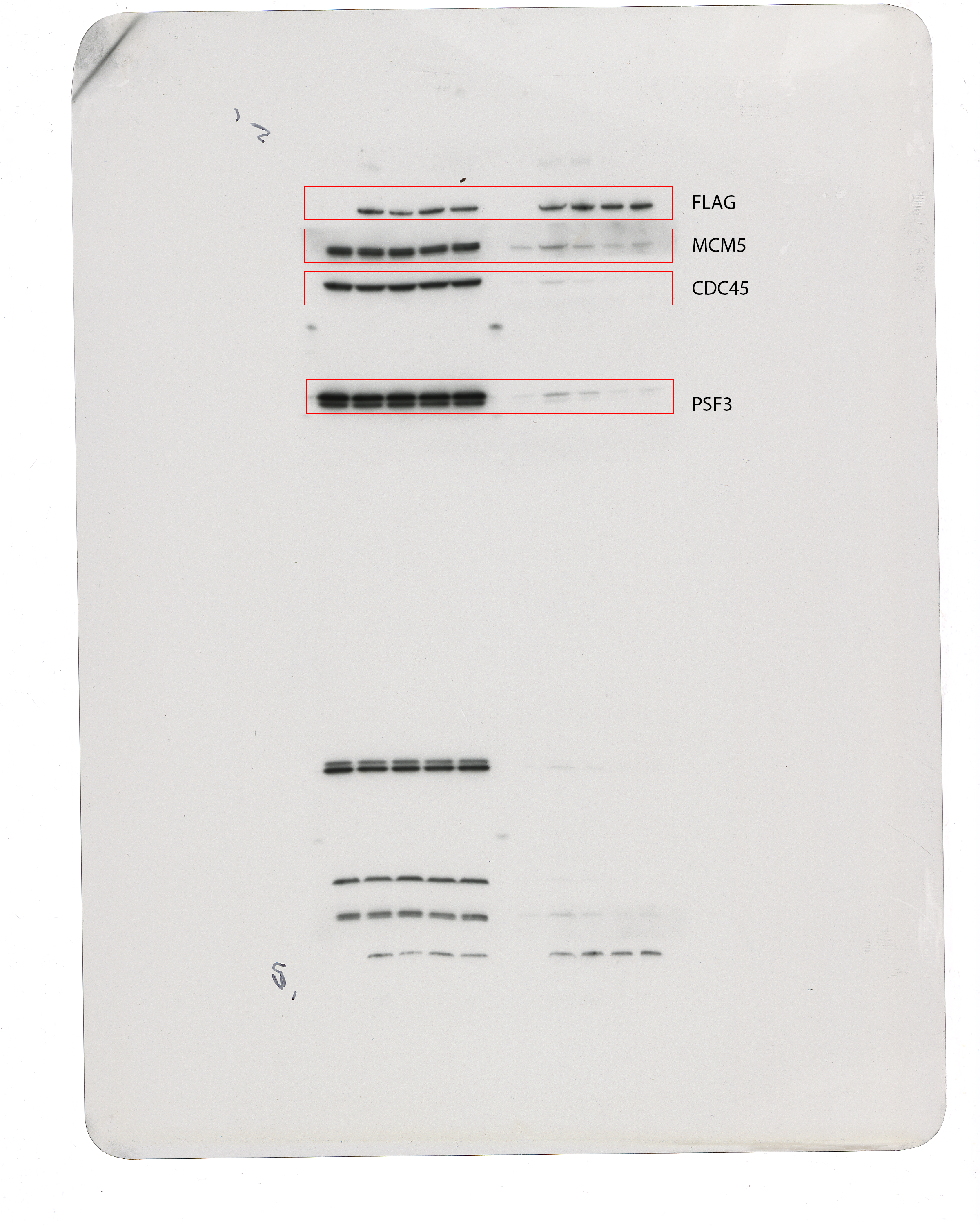

Supplement: Supplementary file 7 — Source data Fig. 7 [file 44318_2024_168_MOESM7_ESM.zip › Figure 7/7B/7B.tif]

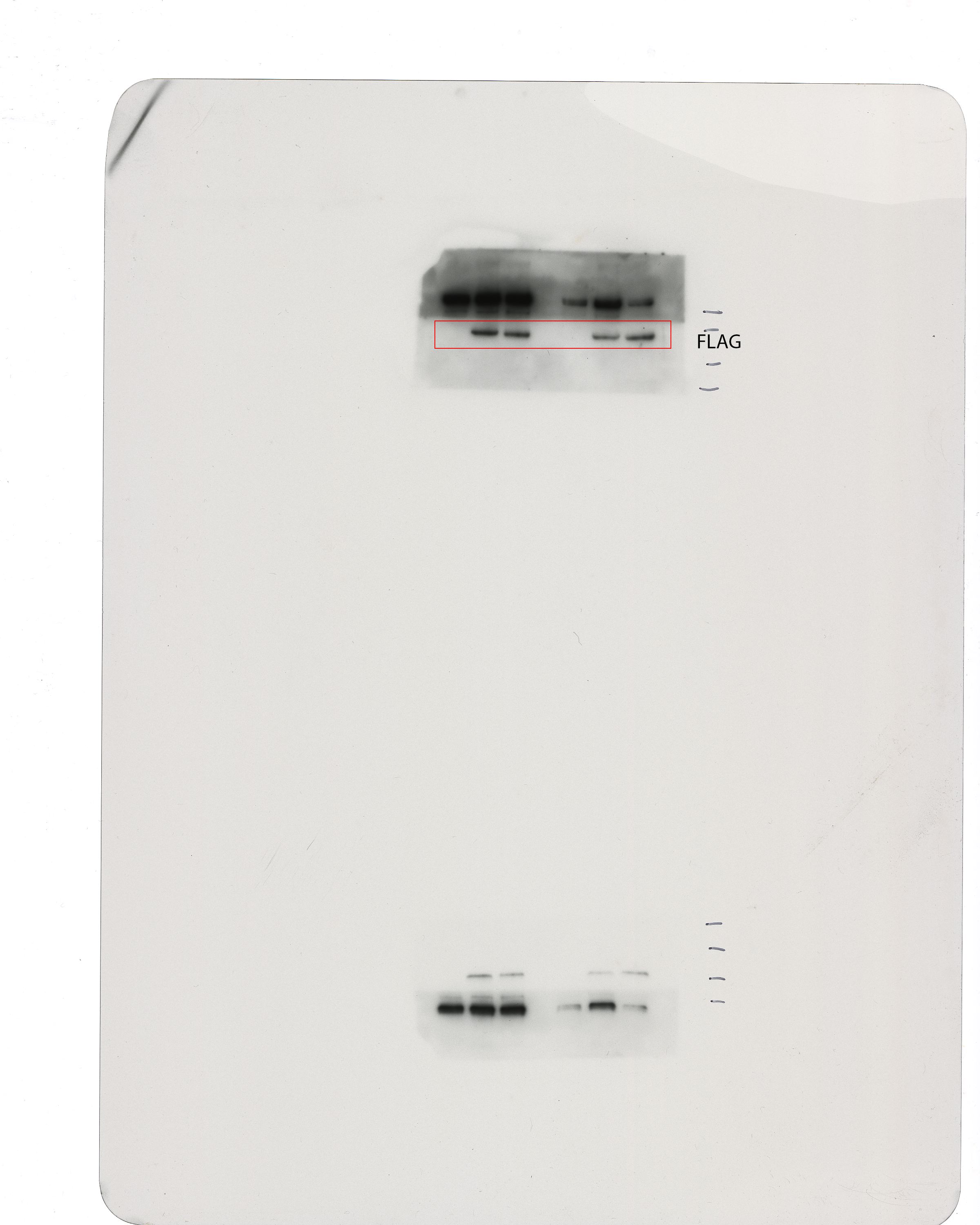

Supplement: Supplementary file 7 — Source data Fig. 7 [file 44318_2024_168_MOESM7_ESM.zip › Figure 7/7A/7A_FLAG.tif]

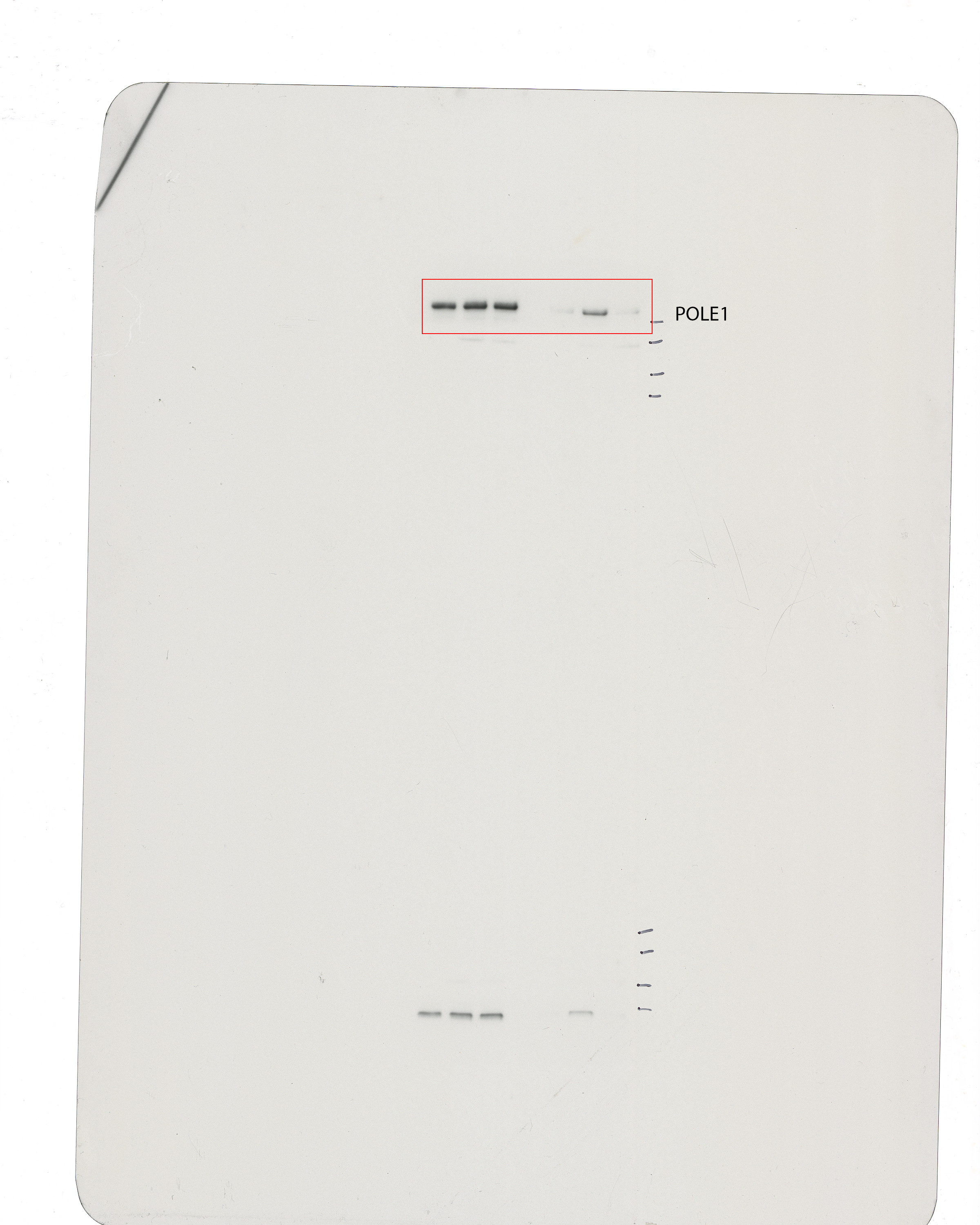

Supplement: Supplementary file 7 — Source data Fig. 7 [file 44318_2024_168_MOESM7_ESM.zip › Figure 7/7A/7A_POLE1.tif]
