# Supplementary material for: A common mechanism for recruiting the Rrm3 and RTEL1 accessory helicases to the eukaryotic replisome
Source: EMBO J. 2024 Jul 22;43(18):3. doi: 10.1038/s44318-024-00168-4 (PMC11405395; doi:10.1038/s44318-024-00168-4)
Supplement: Supplementary file 8 — Expanded View Figures [file 44318_2024_168_MOESM8_ESM.pdf]

Expanded View Figures

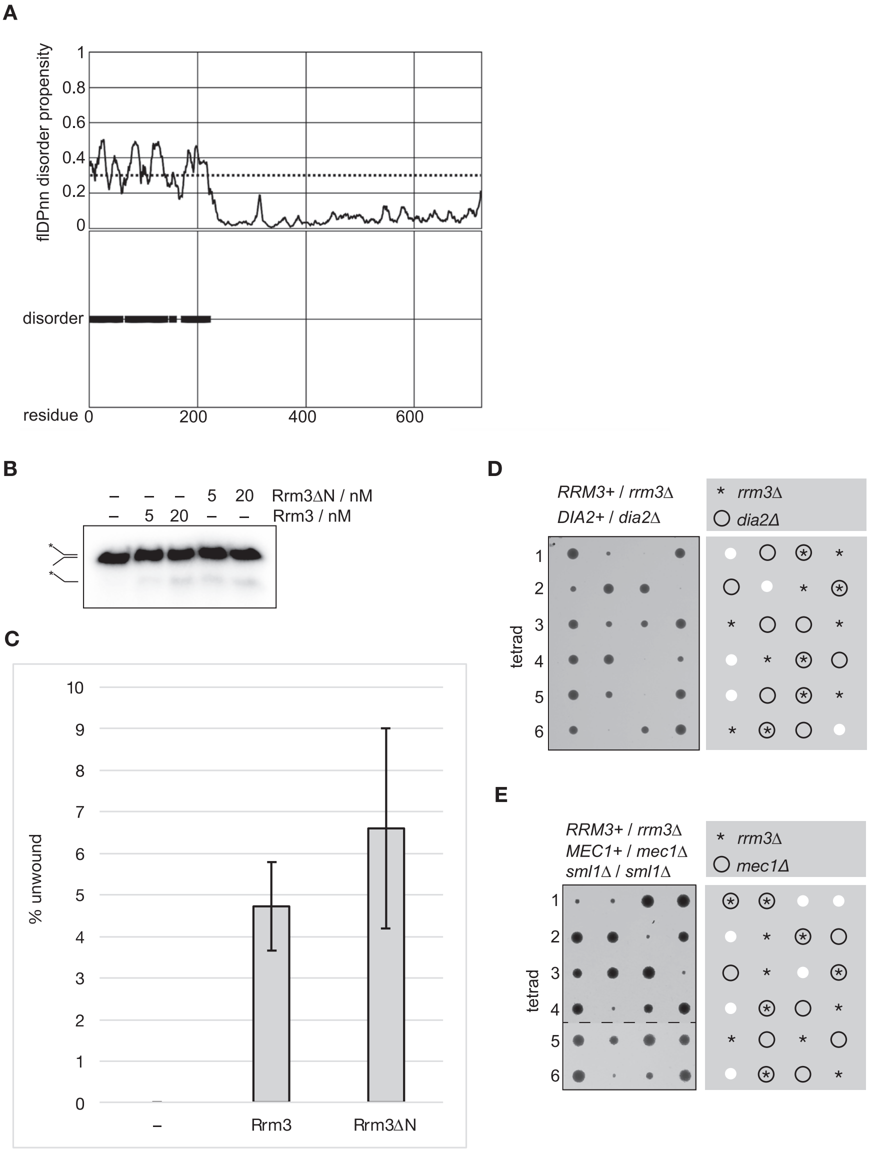

Figure EV1. Characterisation of *rrm3ΔN* mutant in vitro and in vivo.

(**A**) Disorder prediction for *S. cerevisiae* Rrm3, generated using the fIDPnn webserver. Residue numbers are given on the x-axis. (**B**) The ability of Rrm3 and Rrm3ΔN to unwind a 25 bp DNA duplex, formed by annealing oligonucleotide TD254 to TD255, was monitored as described in Methods. * indicates ^{32}P-labelling of TD254. (**C**) Similar experiments to (**B**) were performed three times. The percentage of unwound product was quantified in each case for reactions containing 5 nM of Rrm3, and the figure presents the mean values with standard deviations. (**D**, **E**) Diploid yeast cells of the indicated genotypes were sporulated and the resulting tetrads were then dissected and grown on YPD medium for 2 days at 30 °C.

© The Author(s)

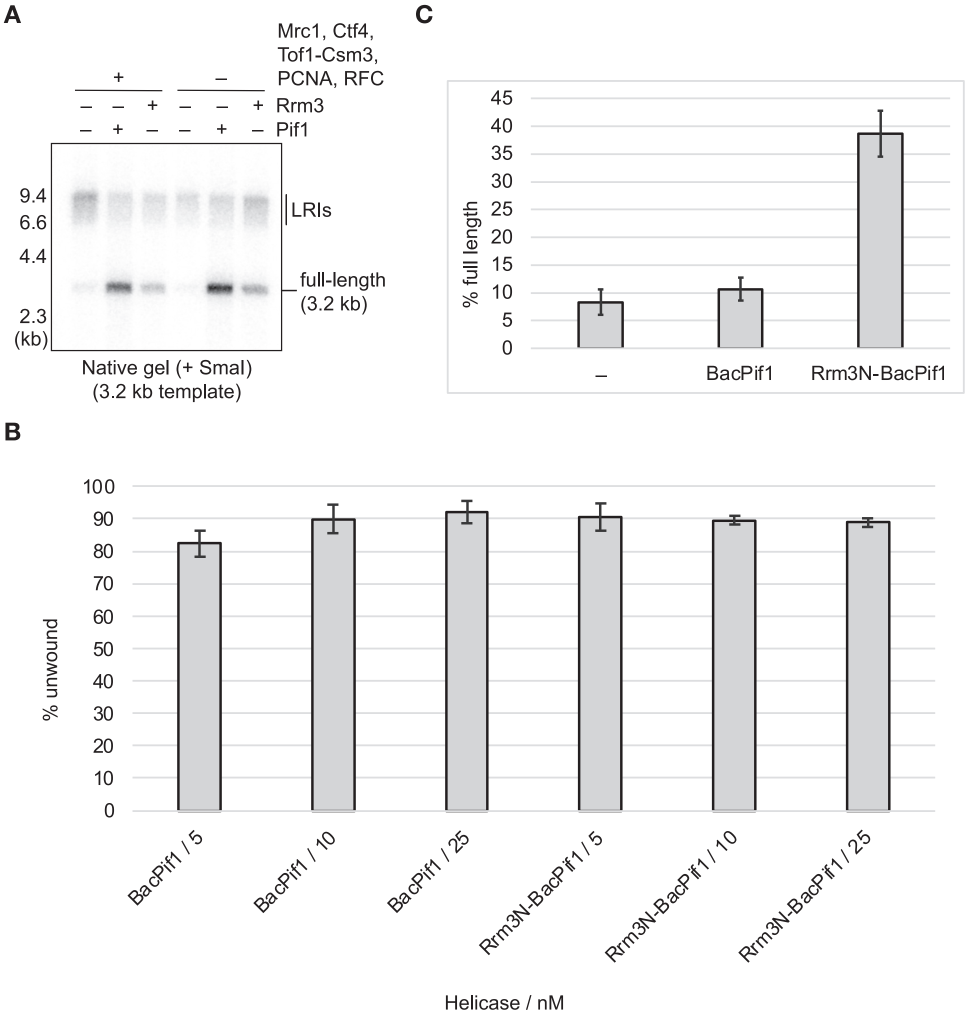

Figure EV2. Supporting data for Fig. 2.

(**A**) A 3189 bp plasmid template (pBS/ARS1WTA) was replicated in the presence or absence of Rrm3 (12.5 nM) or Pif1 (5 nM) and the indicated replisome components. SmaI-digested radiolabelled replication products were resolved in a native agarose gel and detected by autoradiography. (**B**) Similar experiments to Fig. 2C were performed three times. The percentage of unwound product was quantified in each case, and the figure presents the mean values with standard deviations. (**C**) Similar experiments to Fig. 2F were performed three times. The percentage full-length products was quantified in each case, and the figure presents the mean values with standard deviations. Quantification was performed for BacPif1 and Rrm3N-BacPif1 samples that included 5 nM of each helicase.

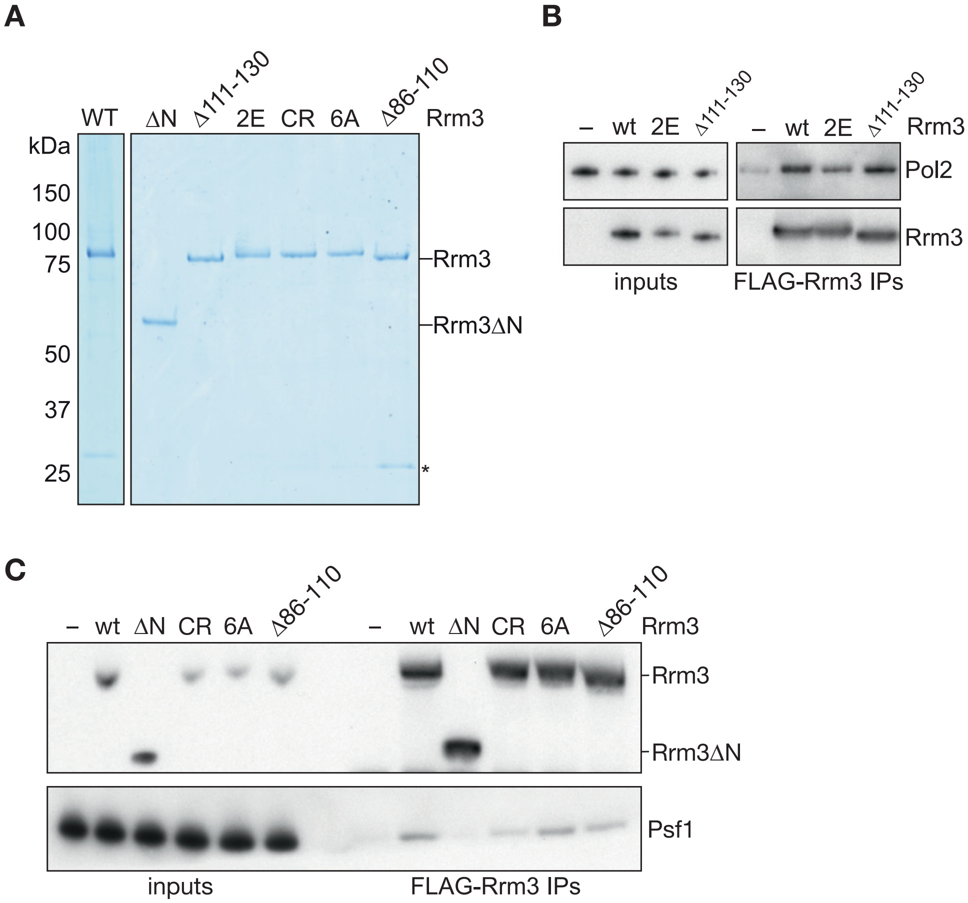

Figure EV3. Generation and characterisation of CMGE-binding mutants of Rrm3.

(**A**) Purified wild-type or mutant versions of Rrm3 visualised by SDS-PAGE and Coomassie staining. * is a contaminating protein. (**B**, **C**) Purified Polε (**B**) or tetrameric GINS complex (**C**) were mixed with FLAG-tagged wild-type Rrm3 or the indicated Rrm3 mutants. Resultant complexes were isolated by anti-FLAG immunoprecipitation and detected by SDS-PAGE and immunoblotting. Rrm3 was detected by anti-FLAG immunoblotting.

 © The Author(s)

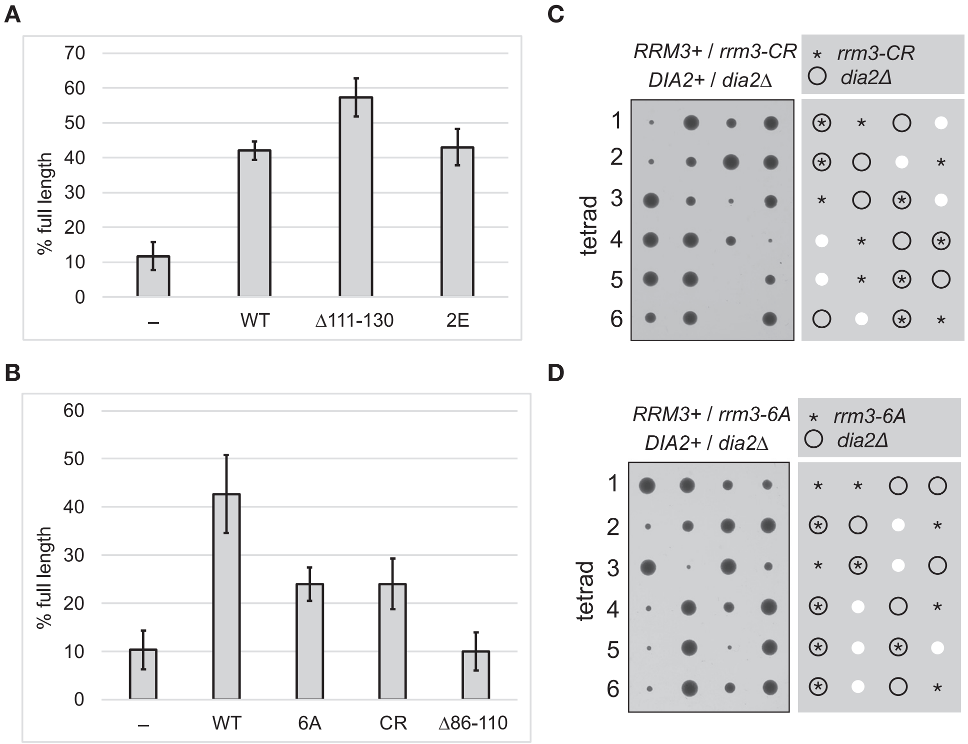

Figure EV4. Supporting data showing that CMGE binding is critical for Rrm3 function.

(**A**, **B**) Similar experiments to Fig. 5A (**A**) and 5B (**B**) were performed three times. The percentage full-length products was quantified in each case, and the figure presents the mean values with standard deviations. (**C**, **D**) Diploid yeast cells of the indicated genotypes were sporulated and the resulting tetrads were then dissected and grown on YPD medium for 2 days at 30 °C.

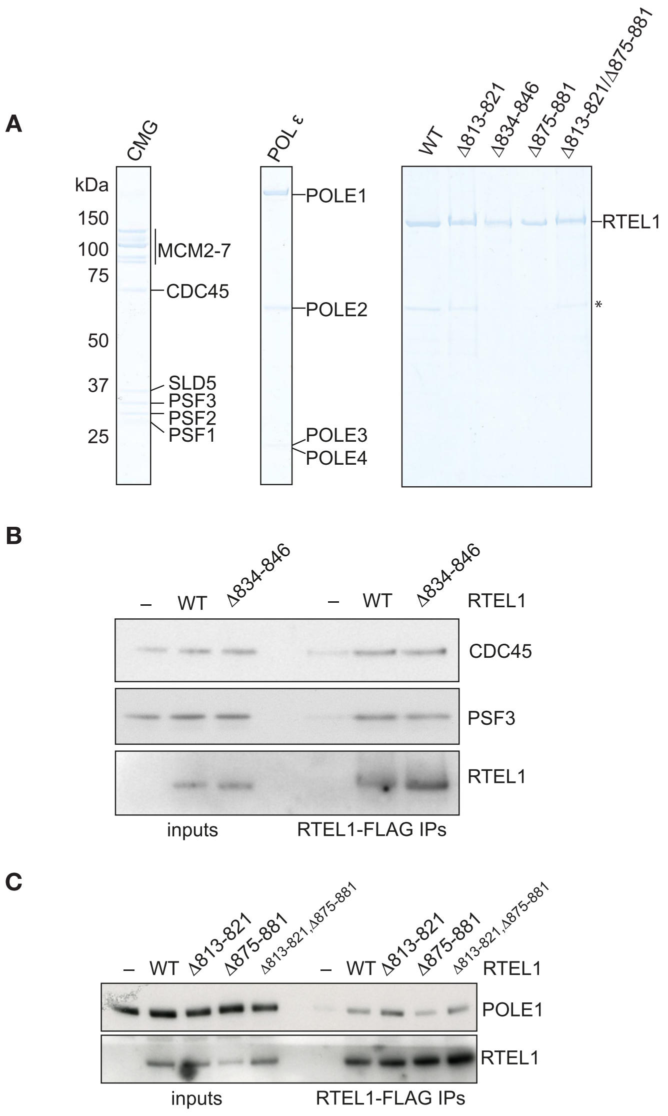

Figure EV5. Generation and characterisation of CMGE-binding mutants of RTEL1.

(**A**) Wild type or mutant versions of *Homo sapiens* RTEL1, CMG and POL ε purified after expression in budding yeast and visualised by SDS-PAGE and Coomassie staining. * indicates a contaminant in purified RTEL1. (**B**, **C**) Purified CMG (**B**) or POL ε (**C**) were mixed with FLAG-tagged wild-type RTEL1 or the indicated RTEL1 mutants. Resultant complexes were isolated by anti-FLAG immunoprecipitation and detected by SDS-PAGE and immunoblotting. RTEL1 was detected by anti-FLAG immunoblotting.

 © The Author(s)
